# Clinical association between current depressive symptoms and odds ratio product in US sleep centers

**DOI:** 10.3389/frsle.2025.1635704

**Published:** 2025-09-12

**Authors:** Archie Defillo, Massimiliano Grassi, Silvia Daccò, Jennifer L. Martin, Veronica Guadagni

**Affiliations:** ^1^Clinical Research and Development Department, Medibio Limited, Savage, MN, United States; ^2^Department of Biomedical Sciences, Humanitas University, Pieve Emanuele, Milan, Italy; ^3^Department of Clinical Neurosciences, Villa San Benedetto Menni Hospital, Hermanas Hospitalarias, Albese con Cassano, Italy; ^4^Personalized Medicine Center for Anxiety and Panic Disorders, Humanitas San Pio X Hospital, Milan, Italy; ^5^VA Greater Los Angeles Healthcare System, University of California, Los Angeles, Los Angeles, CA, United States; ^6^Cerebra Medical, Ltd., Winnipeg, MB, Canada; ^7^Department of Psychology, University of Calgary, Calgary, AB, Canada

**Keywords:** Odds Ratio Product (ORP), current Major Depressive Episode (cMDE), Patient Health Questionnaire (PHQ-9), polysomnography (PSG), sleep architecture, depression

## Abstract

**Introduction:**

The Odds Ratio Product (ORP) is a validated EEG-based measure of sleep depth, more sensitive than traditional metrics. While it has been studied in healthy individuals and those with sleep–wake disorders, its relevance in psychiatric conditions remains unclear. This study examined ORP during sleep and its association with depressive symptoms in a large cohort referred to multiple U.S. sleep centers.

**Methods:**

We retrospectively analyzed data from 829 adults (48.85% female; mean age 43.49 ± 13.74 years) enrolled in two multicenter studies. Each participant completed the Patient Health Questionnaire-9 (PHQ-9) and underwent overnight polysomnography (PSG), with ORP calculated from central EEG channels. Mean and standard deviation ORP values were derived for the full night and Wake, stages 1, 2, 3, and REM sleep. Associations between ORP metrics and depression severity (PHQ-9 total and PHQ-9 ≥10) were tested using linear and logistic regressions, adjusting for age and sex. Model fit was assessed with the Akaike Information Criterion (significance level α = 0.05).

**Results:**

Fixed-effects models outperformed mixed-effects models. Mean ORP during the full night and light sleep (stages 1 + 2) showed a significant U-shaped association with depression, indicating both high and low ORP values relate to greater depressive burden. In stage 3, higher mean ORP was linearly associated with more severe symptoms. Lower ORP variability across the night also correlated with higher depression scores.

**Conclusions:**

ORP shows potential as a non-invasive biomarker for depressive symptoms, with distinct associations depending on sleep depth. Integrating ORP into clinical PSG analyses could improve detection of depression-related sleep patterns.

## 1 Introduction

The Odds Ratio Product (ORP) is a well-established metric used to assess sleep depth and the sleep-wake continuum. ORP provides a continuous, physiologically grounded measure of sleep depth, offering superior sensitivity to both subjective sleep quality and objective sleep architecture, capturing sleep instability and subtle arousal patterns. For example, some studies demonstrated that ORP better predicted sleepiness and sleep fragmentation in obstructive sleep apnea (OSA) patients than standard sleep stage percentages (Younes, 2023). Similarly, other studies found that ORP was more closely associated with subjective sleep quality and daytime functioning in a large community cohort, outperforming traditional metrics like total sleep time and sleep efficiency (Younes et al., 2022). Additionally, studies in insomnia populations have reported that ORP effectively captures increased sleep fragmentation and lighter sleep, correlating strongly with clinical symptoms, whereas conventional measures showed less sensitivity (Riemann et al., 2010). Due to these distinctions, several studies have explored ORP in several sleep disorders, such as idiopathic hypersomnia (Thomas et al., 2022), OSA and its changes during CPAP treatment (Penner et al., 2019), as well as in severe conditions such as OSA-insomnia comorbidity (COMISA; Younes et al., 2021). For this reason, the ORP has been utilized in sleep medicine as an alternative measure of sleep depth, differing from conventional methods recommended by the American Academy of Sleep Medicine (AASM; American Academy of Sleep Medicine, 2023). ORP was first introduced by (Younes et al. 2015) as part of a novel algorithm that quantifies sleep depth on a 0–2.5 scale by analyzing the ratio of power in various EEG frequency bands—particularly delta, theta, alpha, and beta—recorded every 3 s throughout the night. This method differs fundamentally from the AASM scoring rules (American Academy of Sleep Medicine, 2023), which rely on 30-s epochs and categorical stage assignments. The ORP algorithm uses a database of EEG power spectra and applies odds ratio comparisons to determine the likelihood that a given 3-s epoch corresponds to wakefulness vs. deep sleep, yielding a continuous and granular index of sleep propensity. This development was guided by the recognition that traditional staging often fails to capture dynamic changes in brain activity, particularly within stages (e.g., varying depth within N2 sleep), and may overlook micro-arousals or instability that are clinically relevant. Thus, the development of the ORP originated from the need for a more continuous and physiologically relevant measure of sleep depth and arousability than traditional, stage-based scoring. By offering a fine-grained, epoch-by-epoch assessment, ORP enables improved detection of sleep fragmentation, cortical arousability, and stability of the sleep-wake continuum, making it especially valuable in clinical and research contexts where subtle sleep disturbances are present but not detected by standard scoring (Younes, 2023; American Academy of Sleep Medicine, 2023).

Despite the growing application of ORP in healthy individuals and those with sleep–wake disorders, only a few studies have explored its use in psychiatric populations, even though sleep disturbances are highly prevalent and clinically significant in these patients. Psychiatric disorders are well-known to be highly comorbid with sleep disturbances and sleep-wake disorders. In particular, numerous studies have highlighted a strong, bidirectional relationship between depression and sleep disorders (Nutt et al., 2008; Franzen and Buysse, 2008). Depression can cause insomnia, and insomnia can worsen depression symptoms, creating a cyclical pattern (Fang et al., 2019). Notably, individuals diagnosed with insomnia are 10 times more likely to develop depression compared to those with healthy sleep patterns (Nutt et al., 2008; Jindal, 2004). This association has been consistently observed across different age groups, including young, middle-aged, and older adults. The literature consistently reports both subjective and objective alterations in sleep architecture, continuity, and circadian regulation in patients with depression. Patients with major depressive disorder (MDD) frequently experience insomnia symptoms, including prolonged sleep onset latency, increased nocturnal awakenings, early morning awakenings, and reduced total sleep time. These disturbances contribute to perceived poor sleep quality and may exacerbate mood symptoms (Nutt et al., 2008). Polysomnographic studies have identified specific alterations in sleep architecture associated with depression. These include a reduction in slow-wave sleep (SWS), which reflects diminished restorative sleep, and shortened REM sleep latency, characterized by earlier onset of rapid-eye-movement (REM) sleep after sleep initiation. Additionally, patients often exhibit increased REM density—a higher frequency of rapid eye movements during REM sleep—which has been linked to emotional dysregulation and may serve as a biological marker for depression (Fang et al., 2019; Baglioni et al., 2016; Riemann et al., 2020). However, the interpretation of sleep architecture findings in depression is constrained by several important factors. Depression is a clinically heterogeneous condition, and its sleep-related manifestations can vary depending on subtype, symptom severity, and treatment status (American Psychiatric Association, 2022). Moreover, there is substantial overlap between the sleep architecture associated with depression and that seen in co-occurring sleep disorders, including obstructive sleep apnea (OSA), insomnia, restless legs syndrome (RLS), and periodic limb movement disorder (PLMD; Lee et al., 2019). These sleep conditions independently disrupt sleep continuity and structure, leading to increased arousals, reduced slow-wave sleep, altered REM dynamics, and overall sleep fragmentation (Wang et al., 2025). Such overlapping features make it challenging to attribute specific polysomnographic findings solely to depression. The effects of antidepressant medications, which commonly suppress REM sleep and alter NREM patterns (Steiger and Pawlowski, 2019) further complicate interpretation. In addition, the often-observed discrepancy between subjective sleep complaints and objective measures in depression suggests that traditional sleep metrics may not adequately capture the full extent of sleep disruption. This highlights the need for more sensitive and physiologically grounded indices, such as the ORP, which may better reflect both objective sleep instability and perceived sleep quality, serving as a novel and sensitive biomarker for detecting sleep characteristics associated with depressive symptoms in patients with comorbid sleep-wake disorders.

Early detection of depression are essential for achieving full remission and improving long-term outcomes, including reducing the risk of recurrence, chronicity, and significant functional impairment (Perna et al., 2020, 2021). Consistent with this, strong evidence supports the effectiveness of programs that integrate depression screening with appropriate follow-up interventions in improving clinical outcomes among adults (O'Connor et al., 2009; Siniscalchi et al., 2020; Siu et al., 2016).

Among available tools, the Patient Health Questionnaire-9 (PHQ-9; Kroenke and Spitzer, 2002; Kroenke et al., 2001; Spitzer et al., 1999) is considered one of the most reliable and widely adopted depression screening instruments. It offers high sensitivity and specificity for major depressive disorder, particularly in primary care (Maurer et al., 2018). Grounded in DSM-5 criteria, the PHQ-9 allows for both symptom severity assessment and provisional diagnosis. Its clinical utility and diagnostic accuracy have led to its endorsement by the United States Preventive Services Task Force (USPSTF) and other expert bodies for routine use in both primary and specialty care (O'Connor et al., 2009; Siniscalchi et al., 2020; Maurer et al., 2018; Lichtman et al., 2008; Costantini et al., 2021).

Due to its brevity, psychometric strength, and ease of administration, the PHQ-9 is particularly well-suited for research exploring the relationship between depressive symptoms and physiological parameters such as sleep architecture.

Despite clear recommendations, a substantial gap persists between the need for depression treatment and its actual delivery worldwide. Physician adherence to routine depression screening remains limited (O'Connor et al., 2009) and depressive symptoms are frequently underrecognized in patients attending outpatient specialty clinics (Wang et al., 2017). For example, unrecognized depression has been reported in 45 to 51% of medical patients and in ~54% of surgical patients (Rahman et al., 2015).

This underdetection is particularly concerning in specialty settings such as SCs, where patients typically undergo PSG to investigate sleep disturbances. Given the previously discussed bidirectional relationship between sleep and depression, systematic screening in these settings is especially warranted. However, barriers such as time limitations, lack of standardized protocols, and insufficient integration of mental health evaluation into sleep medicine workflows likely contribute to this gap. As a result, depressive symptoms often go unrecognized in patients undergoing evaluation for sleep-wake disorders.

To date, no studies have specifically investigated the relationship between the ORP and depressive symptoms. Before ORP can be considered a potential biomarker for depression in sleep medicine, it is essential to first understand whether and how ORP correlates with depressive symptom severity. Therefore, the present study aims to evaluate ORP values across different sleep stages and examine their linear and non-linear associations with depressive symptoms, as measured by the PHQ-9, in a large cohort of patients referred for sleep-wake disorder evaluation at multiple SCs across the United States. This foundational analysis is a critical step toward establishing whether ORP can serve as a sensitive, objective indicator of depressive symptomatology in clinical sleep populations.

If such associations are confirmed, ORP may serve as the foundation for an automated, physiology-based screening tool that passively identifies individuals at risk for depression during routine polysomnographic assessments. This approach could overcome key limitations of current screening practices by integrating mental health detection into existing sleep study protocols without requiring additional time or specialized clinical training.

## 2 Methods

### 2.1 Sample characteristics

This retrospective analysis is based on data collected in the context of two prior studies. The Sleep Analysis of Depressive Burden (SADB) study (ClinicalTrials.gov Identifier: NCT04232267) and the Sleep Signal Analysis for Current Major Depressive Episode (SAMDE) study (ClinicalTrials.gov Identifier: NCT05708222) are both cross-sectional, naturalistic, single-arm, multicenter trials sponsored by Medibio Ltd (Minnesota, USA). Participant recruitment for SADB occurred from December 2019 to February 2022 across multiple U.S. sites, including sleep clinics in Ohio and Minnesota, while SAMDE recruitment took place from June 2023 to July 2024 across additional sites in Texas, North Carolina, South Carolina, Florida, Minnesota, and Ohio. Inclusion criteria encompassed adults aged 18–75 (SADB) and 22–75 (SAMDE), able to provide informed consent and adhere to study procedures. Exclusion criteria differed slightly, with SADB excluding active substance abuse and SAMDE excluding pacemaker recipients, heart transplant patients, and those undergoing CPAP titration studies. Psychotropic medication use was permitted to enhance sample representativeness. Preliminary findings on depression prevalence in both cohorts have been reported in earlier publications (Daccò et al., 2023, 2025).

### 2.2 Harmonized protocol

Both SADB and SAMDE studies adopted a harmonized protocol to ensure data comparability across sites and studies. Upon enrollment, participants completed self-administered forms capturing demographic and clinical variables, followed by the PHQ-9 to assess depressive symptom severity. Additionally, SAMDE participants completed a fully structured diagnostic interview using the Mini International Neuropsychiatric Interview for major depressive episode diagnosis (Sheehan et al., 1998).

### 2.3 PSG measures and sleep variables

PSG recordings in both studies adhered strictly to AASM guidelines (American Academy of Sleep Medicine, 2023), utilizing identical EEG derivations including six recommended channels (F4-A1, C4-A1, O2-A1) and backup channels (F3-A2, C3-A2, O1-A2). These standardized recording protocols and sleep staging criteria were applied uniformly, enabling reliable pooling and comparative analyses of sleep variables relevant to depressive burden.

### 2.4 Covariates

Collected covariates included age, sex, medical history, comorbidities, and current medications, allowing control for confounding factors during analyses. The consistent methodology across multiple U.S. clinical sites—including those overlapping between SADB and SAMDE in Minnesota and Ohio—facilitated robust, generalizable insights into the relationship between sleep characteristics and depression.

### 2.5 PHQ-9

The PHQ-9 (Kroenke and Spitzer, 2002; Kroenke et al., 2001) is a validated 9-item self-report questionnaire widely used in depression assessment (Maurer et al., 2018). Its 9 items align with the 9 DSM-5 criteria for a Major Depressive Episode (American Psychiatric Association, 2022). Item response options for each item range from “not at all” (score of 0) to “several days” (score of 1), “more than half the days” (score of 2), and “nearly every day” (score of 3), reflecting how often each symptom has bothered the respondent over the past 2 weeks. The PHQ-9 total score for the nine items ranges from 0 to 27. The scores 5, 10, 15, and 20 represent the cutoffs for mild, moderate, moderately severe, and severe depression symptoms, respectively.

A cutoff of 10 or greater is a widely used threshold to screen for current Major Depressive Episode (cMDE). A recent and comprehensive Individual Participant Data Meta-analysis (Negeri et al., 2021), including approximately 44,500 participants, evaluated the accuracy of PHQ-9 to detect a cMDE through comparisons with the reference standard. Compared to reference standards, sensitivity and specificity for a cut-off of ≥ 10 ranged from 0.67 to 0.88 and from 0.86 to 0.88, respectively. Specifically, compared to semi-structured psychiatric diagnostic interviews, sensitivity and specificity for a cutoff of ≥ 10 (95% CI) were 0.88 (0.82–0.92) and 0.86 (0.82–0.88). For fully structured interviews, sensitivity and specificity ranged from 0.67 (0.57–0.76) to 0.75 (0.66–0.82), and from 0.86 (0.80–0.90) to 0.88 (0.84–0.91). Overall, PHQ-9 demonstrated satisfactory accuracy in depressive episode detection. Consistently, a score of ≥ 10 has been associated with an increased risk of major depression more than 2.6 times (Kroenke et al., 2001). Overall, based on the large body of scientific evidence concerning PHQ-9, this cutoff threshold (≥ 10) approach is advised as the most reliable for screening use in clinical practice and clinical trials (He et al., 2020).

### 2.6 Sleep staging

Sleep staging was performed using an automated system implemented via the STAGER software (Medibio Limited, Savage, MN, USA). This tool processes polysomnographic recordings stored in EDF format by focusing on EEG signals from six pre-selected channels. The recordings are first segmented into 30-s epochs, during which a detailed spectral analysis is performed to compute absolute and relative power values across key frequency bands. These features are then input into a pipeline of machine learning and deep learning algorithms. Initially, a convolutional neural network and gradient-boosting machine learning algorithms are used, followed by two additional temporal-aware models, such as recurrent neural networks. An ensemble method integrates the outputs from these classifiers to assign each epoch to one of the five standard sleep stages (wake, N1, N2, N3, and REM) in accordance with the American Academy of Sleep Medicine (AASM) guidelines (American Academy of Sleep Medicine, 2023). Prior to analysis, the software requires the entry of lights-off and lights-on times to accurately delineate the sleep period. The performance of STAGER has been validated in a recent study (Grassi et al., 2023). In 40 clinical PSGs, STAGER's automatic staging achieved an overall percentage agreement of 83.8% (95 % CI = 82.3%−85%) vs. the majority vote of three AASM-certified technicians, with a Cohen's κ of 0.78 (substantial agreement). Stage-specific positive-percent-agreement was 90.7% for REM, 89.7% for N2, 87.3% for N3, 81.6% for Wake, and 51% for N1; except for a modest drop in Wake sensitivity, these values were statistically comparable to or higher than those obtained by individual human scorers.

### 2.7 Odds ratio product

The odds ratio product (ORP) is a continuous metric that quantifies sleep depth and wake propensity on a scale from 0 (indicative of very deep sleep) to 2.5 (reflecting full wakefulness). Unlike conventional sleep staging, which classifies sleep in discrete 30-s epochs, ORP is computed every 3 s from EEG recordings, allowing for a much more refined temporal resolution of sleep dynamics (Younes, 2023). In brief, the method initially applies a fast Fourier transform to brain wave bands from 0.33 Hz to 60 Hz in non-overlapping 3-s epochs. Then, the power across four frequency bands (Alpha 7.3–14.0 Hz, Beta 14.3–35.0 Hz, Delta 0.3–2.3 Hz, and Theta 2.7–6.3 Hz) is evaluated. For each band, the power is ranked into deciles based on a normative dataset derived from a broad array of clinical polysomnograms. These decile scores are then concatenated to form a unique four-digit “bin” number for each epoch. A lookup table, constructed from the frequency of these patterns during wake and arousal, converts the bin number into a probability that is normalized (by dividing by 40) to yield an ORP value between 0 and 2.5.

This continuous measure enables the detection of subtle transitions between sleep and wake states that are not discernible with traditional, categorical staging. In addition, because ORP captures variations in sleep depth within the same conventional stage, it provides an enriched depiction of the sleep architecture. For example, a strong association between ORP values and the probability of spontaneous arousal in the subsequent epoch has been found, underscoring its validity as a sensitive indicator of sleep stability and depth (Younes et al., 2015). ORP can be graphically displayed as an epoch-by-epoch trace across the night, or summarized as average values within traditional sleep stages, and even as the percentage of total recording time spent in defined ORP ranges. These features offer significant advantages, particularly in clinical research, where they can help identify subtle sleep abnormalities that may underlie various sleep disorders or predict therapeutic outcomes.

In our study, the ORP value was computed for each non-overlapping 3-s epoch throughout the entire polysomnographic recording, from lights-off to lights-on, using the central EEG channels (C4-A1 and C3-A2) among the six channels recommended by the AASM guidelines (Younes, 2023). These computations were performed via the API provided by Cerebra Medical LTD. (Winnipeg, CA).

For the entire polysomnographic recording, the mean ORP and its standard deviation were computed and used in the statistical analyses. Additionally, the mean and standard deviation of ORP for each sleep stage (Wake, N1 + N2, N3, and REM) were also calculated and incorporated into the analyses. N1 has been incorporated into N2 (sometimes collectively referred to as light sleep). Algorithmically, despite merging N1 and N2, the STAGER software can differentiate each stage by identifying the presence of spindle bands and K-complex.

Epochs in which the Cerebra API identified problems in the EEG signal (e.g., artifacts, noise, lack of signal) that prevented a correct ORP calculation, as well epochs the STAGER software staged as non-classified (U) for similar problems in the EEG signal, were excluded from the analysis.

### 2.8 Statistical analysis

We conducted statistical analyses to assess the associations between the mean and standard deviation of ORP during the whole PSG and two depression outcome measures: the total PHQ-9 score (continuous variable), and a dichotomized PHQ-9 score based on the conventional cut-off of 10 (that is, ≥10). We applied linear regression for the continuous PHQ-9 score and logistic regression for the PHQ-9 cut-off of 10.

To account for potential non-linear relationships, both linear and quadratic terms for the ORP variables were included in the analyses. Age and sex were included as covariates in all models to adjust for potential confounding effects and isolate the residual association between ORP and depression measures. The independent variables were mean-centered prior to model fitting, and quadratic terms were computed after centering. This approach reduces multicollinearity between the linear and quadratic terms and enhances the interpretability of regression coefficients of the ORP variables.

If a subject never exhibited a particular sleep stage or if EEG signal problems during certain epochs made it impossible to calculate the ORP, then the ORP variables for that sleep stage were deemed uncalculable for that individual. Consequently, these instances were excluded from the analysis of the ORP variable for those epochs. As a result, the overall number of epochs for certain sleep stages may be smaller than the total sample size used in the analysis of the entire PSG.

Data were collected from multiple sleep centers, each of which potentially differed in equipment, protocols, and patient characteristics. Therefore, we evaluated both fixed-effects and mixed-effects models to determine if center-related variability should be incorporated into our analyses. To this end, we developed a grouping variable by aggregating centers based on their state location and management by the same organization, as centers under the same organization typically followed similar protocols and utilized the same PSG system. One exception was a Texas center (TX) where a subset of PSG recordings was performed using an alternative system; these recordings were assigned a distinct level within the grouping variable (TA). Because both SADB and SAMDE employed naturalistic recruitment and shared similar protocols, the study of origin was not treated as a separate factor in this grouping.

For each independent-dependent variable combination, we evaluated four model structures: 1) Fixed-effects model (no random effects); 2) Mixed-effects model with a random intercept; 3) Mixed-effects model with a random intercept and a random slope for the independent variable; 4) Mixed-effects model with random effects for the intercept, independent variable, and covariates. All mixed-effects models were initially fitted using Maximum Likelihood (ML) to ensure comparability between models, including the fixed-effects model. For mixed-effects models with more than one random effect (models 3 and 4), we tested three different covariance structures for the random effects: independent, diagonal, and full covariance matrix. Model selection was guided by the Akaike Information Criterion (AIC), which balances model complexity against goodness-of-fit by penalizing unnecessary parameters. The model with the lowest AIC was considered the most parsimonious and best-fitting for each independent-dependent variable pairing. If a mixed-effects model was selected as the best model, it was refitted using Restricted Maximum Likelihood (REML) to obtain more accurate estimates of variance components.

To assess the association between each ORP independent variable and the total PHQ-9 score (linear regression), statistical significance was evaluated using the *t*-test for the regression coefficient. The regression coefficient itself was used to quantify the direction of the association, while the standardized regression coefficient (β coefficient) was computed to facilitate the interpretation of the magnitude of the effect. We also quantified the additional variance explained by the ORP terms as the change in the coefficient of determination (Δ*R*^2^), calculated by subtracting the *R*^2^ of the covariate-only model from the *R*^2^ of the full model that included both covariates and the two ORP terms (Δ*R*^2^ = *R*^2^_full_ – *R*^2^_covariates_).

For the association between each ORP independent variable and the PHQ-9 cut-off 10 outcomes (logistic regression), statistical significance was assessed using the z-test for the regression coefficient. Also in this analysis, the regression coefficient itself was used to quantify the direction of the association, while the standardized regression coefficient (β coefficient) was computed to facilitate the interpretation of the magnitude of the effect. We also assessed the gain in discriminative ability by computing the change in the area under the receiver-operating characteristic curve (ΔAUC), defined as the difference between the AUC of the full model (covariates + ORP terms) and that of the covariate-only model.

All statistical analyses were performed using Python (version 3.12.1; Python Software Foundation, 2024). Statistical significance was set at α = 0.05.

## 3 Results

### 3.1 Descriptive statistics

**Figure 1:**
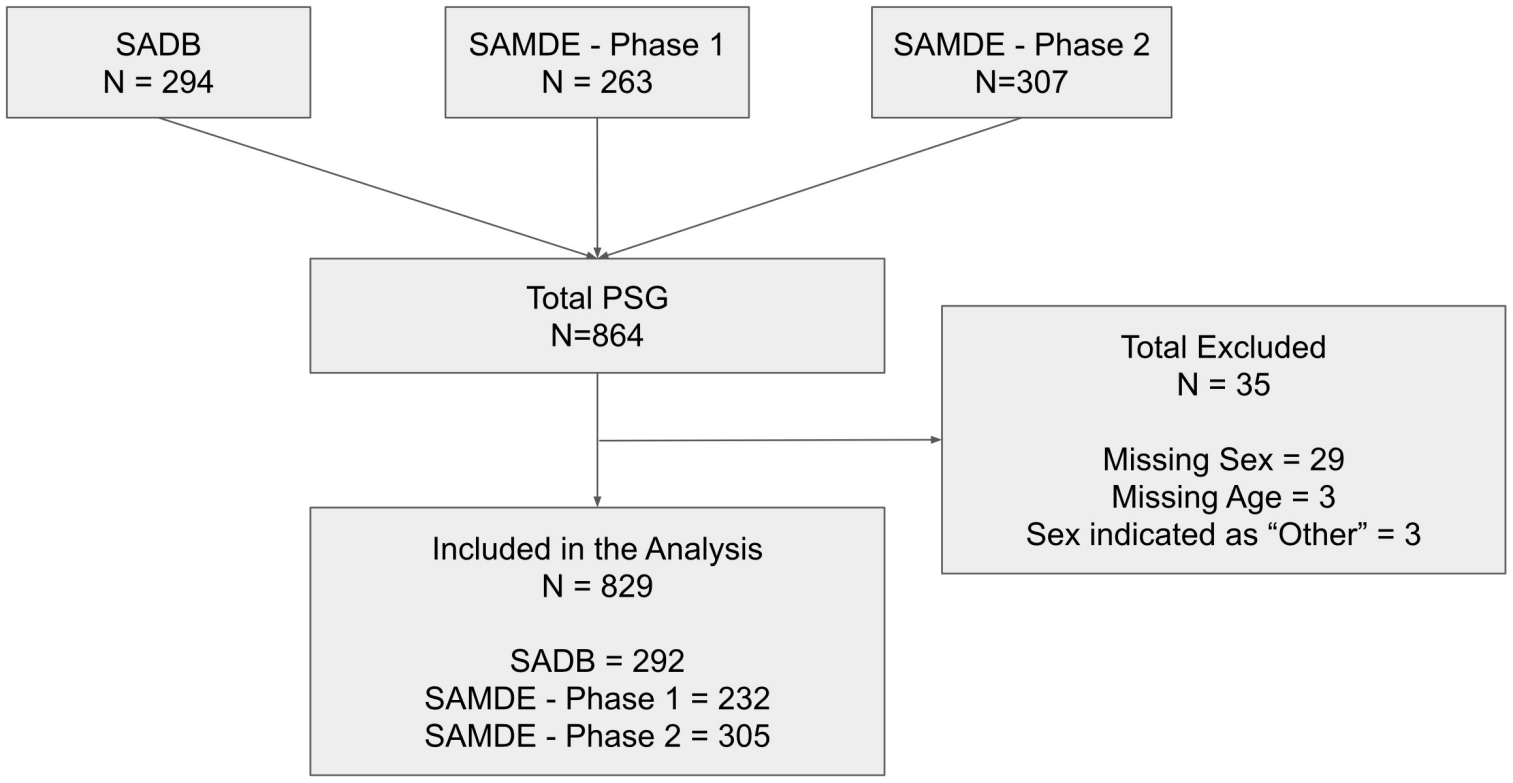
Participant selection flow diagram.

Of the 864 PSG initially available for this study, 829 were finally included in the analysis (292 from the SADB study, 232 from the SAMDE Phase 1 study, and 305 from the SAMDE Phase 2 study; Figure 1). Thirty-two were excluded from the analysis because the Sex was not provided by the subject (29 from the SAMDE Phase 1 study) or was indicated by the subjects as “other” (2 from the SAMDE Phase 2 study and 1 from the SAMDE Phase 1 study). Moreover, three additional subjects were excluded because age was missing (2 from the SADB study and 1 from the SAMDE Phase 1 study). Descriptive statistics for the entire sample are summarized in Table 1.

**Table 1:** Descriptive statistics for full sample.

**Property**	**Total sample (*****n*** = **829)**	
	**Mean/number** ^§^	**std dev/%** ^§^
Age (years)	43.49	13.74
Females	424^§^	48.85^§^
Body mass index (BMI)	32.40	8.87
BMI, underweight and healthy weight (BMI of 24.9 or less)	149^§^	16.04^§^
BMI, overweight (BMI of 25–29.9)	232^§^	17.97^§^
BMI, obesity (BMI of 30–39.9)	315^§^	38.00^§^
BMI, severe obesity (BMI of 40 or more)	133^§^	27.99^§^
Total sleep time (hours)	5.47	1.20
Apnea-hypopnea Index	15.64	20.53
Sleep efficiency	79.24	14.48
SpO_2_ < 88% (percentage)	4.24%	11.29%
Respiratory arousal index	11.71	16.13
Periodic leg movement arousal index	1.30	3.31
Spontaneous arousal index	7.84	6.08
Total arousal index	21.14	16.94
PHQ-9 total score	8.17	5.75
avg ORP all stages	1.27	0.31
avg ORP N1 + N2	1.12	0.30
avg ORP N3	0.60	0.26
avg ORP REM	1.41	0.36
avg ORP wake	2.17	0.18
std dev ORP all stages	0.57	0.12
std dev ORP N1 + N2	0.40	0.08
std dev ORP N3	0.21	0.07
std dev ORP REM	0.26	0.07
std dev ORP wake	0.20	0.08

Avg, average; N1, Non-REM Sleep Stage 1; N2, Non-REM Sleep Stage 2; N3, Non-REM Sleep Stage 3; ORP, Odds Ratio Product; PHQ-9, Patient Health Questionnaire, 9 items (major depression module); REM, Rapid Eye Movement; std dev, standard deviation; ^§^Values indicate frequencies and percentages.

Participants were grouped into seven distinct groups based on their recruitment centers, which have been used in the random-effect models: Minnesota (MN) with 262 subjects, South Carolina (SC) with 202 subjects, Texas (TX) with 121 subjects, North Carolina (NC) with 113 subjects, Ohio (OH) with 107 subjects, the alternative PSG machine in Texas (TA) with 14 subjects, and Florida (FL) with 10 subjects. Descriptive statistics stratified by recruitment center are summarized in Supplementary Tables S2 and S3 in the Supplementary materials.

Descriptive statistics for the entire sample and stratified by recruitment center are summarized in Table 1.

### 3.2 Assessing the role of age and sex as covariates in the ORP-depression association

To assess the appropriateness of including age and sex as covariates, we first examined their association with depressive symptoms. In all models tested—including linear regression models using the PHQ-9 total score as the dependent variable and logistic regression models using a dichotomized PHQ-9 cutoff of 10—both age and sex were significantly associated with depression outcomes (all *p*-values < 0.001). Age was negatively associated with depressive symptoms, indicating a decrease in symptom severity with increasing age. Furthermore, female participants exhibited higher levels of depressive symptoms compared to males. Full results are available in the Supplementary materials (Supplementary Tables S4–S9).

### 3.3 Model selection and center effects

The analysis examined the associations between the mean and standard deviation of ORP during the whole PSG and depression severity, as measured by the PHQ-9 total score (linear regression) and the dichotomized PHQ-9 based on a conventional cut-off score of 10 (logistic regression). Linear regression models were applied to the continuous PHQ-9 score, while logistic regression models were used for the dichotomized PHQ-9 outcome.

For all analyses, model selection based on the Akaike Information Criterion (AIC) consistently favored the fixed-effects model over the mixed-effects alternatives. Despite data being collected from multiple sleep centers, the variability between centers did not seem to substantially influence the associations between ORP and PHQ-9 outcomes. The lack of a significant center effect indicates that the relationships observed are robust across different clinical settings and are unlikely to be driven by site-specific differences in equipment, protocols, or patient populations. Complete results, including the AIC values for all models, are provided in the Supplementary materials (Supplementary Tables S6 and S7).

### 3.4 Association of PHQ-9 with mean ORP

**Figure 2:**
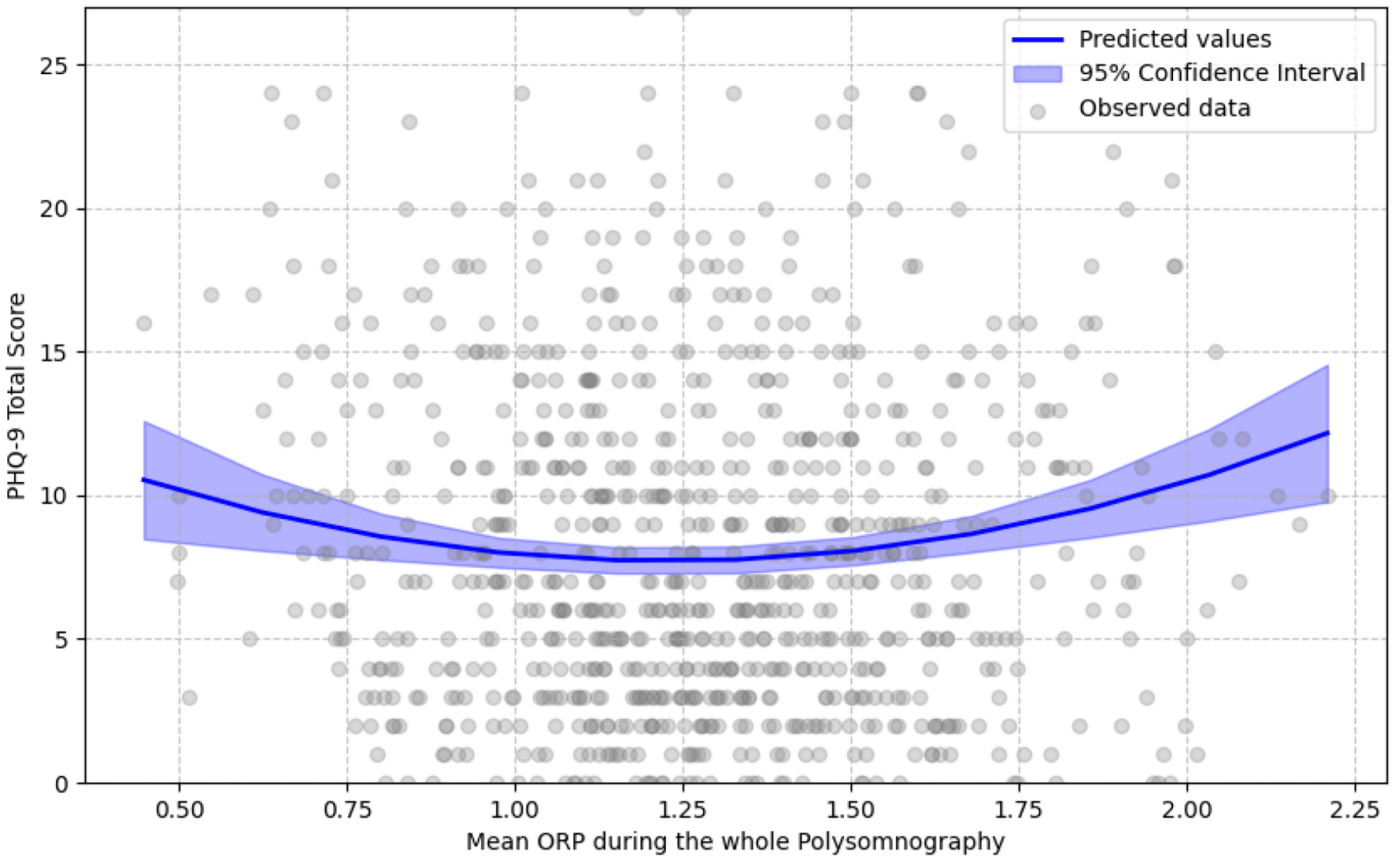
U-shaped relationship of mean ORP in whole polysomnography with PHQ-9 score. Gray circles show each participant's mean ORP across the entire polysomnography (0 = very deep sleep, 2.5 = full wakefulness) against their total PHQ-9 score. The solid blue line represents values predicted by the best-fitting age- and sex-adjusted fixed-effects quadratic regression; the translucent band is the 95% confidence interval. The linear term was not statistically significant, whereas the quadratic term was positive and significant, yielding a U-shaped curve. This indicates that both higher (shallower sleep) and lower (deeper sleep) mean ORP values are associated with higher depressive-symptom burden.

For the linear regression models, the mean ORP during the whole PSG (from light-off to lights-on) showed no significant linear association with PHQ-9 total scores (β = 0.022, *p* = 0.521). However, a significant positive quadratic relationship was detected (β_quadratic_ = 0.078, *p* = 0.001), suggesting that the relationship follows a U-shaped or convex pattern (Figure 2). Since the variables were mean-centered, this indicates that individuals with either higher (shallower sleep) or lower (deeper sleep) mean ORP values across the night were more likely to experience higher depression scores than individuals with average mean ORP values during the whole PSG.

**Figure 3:**
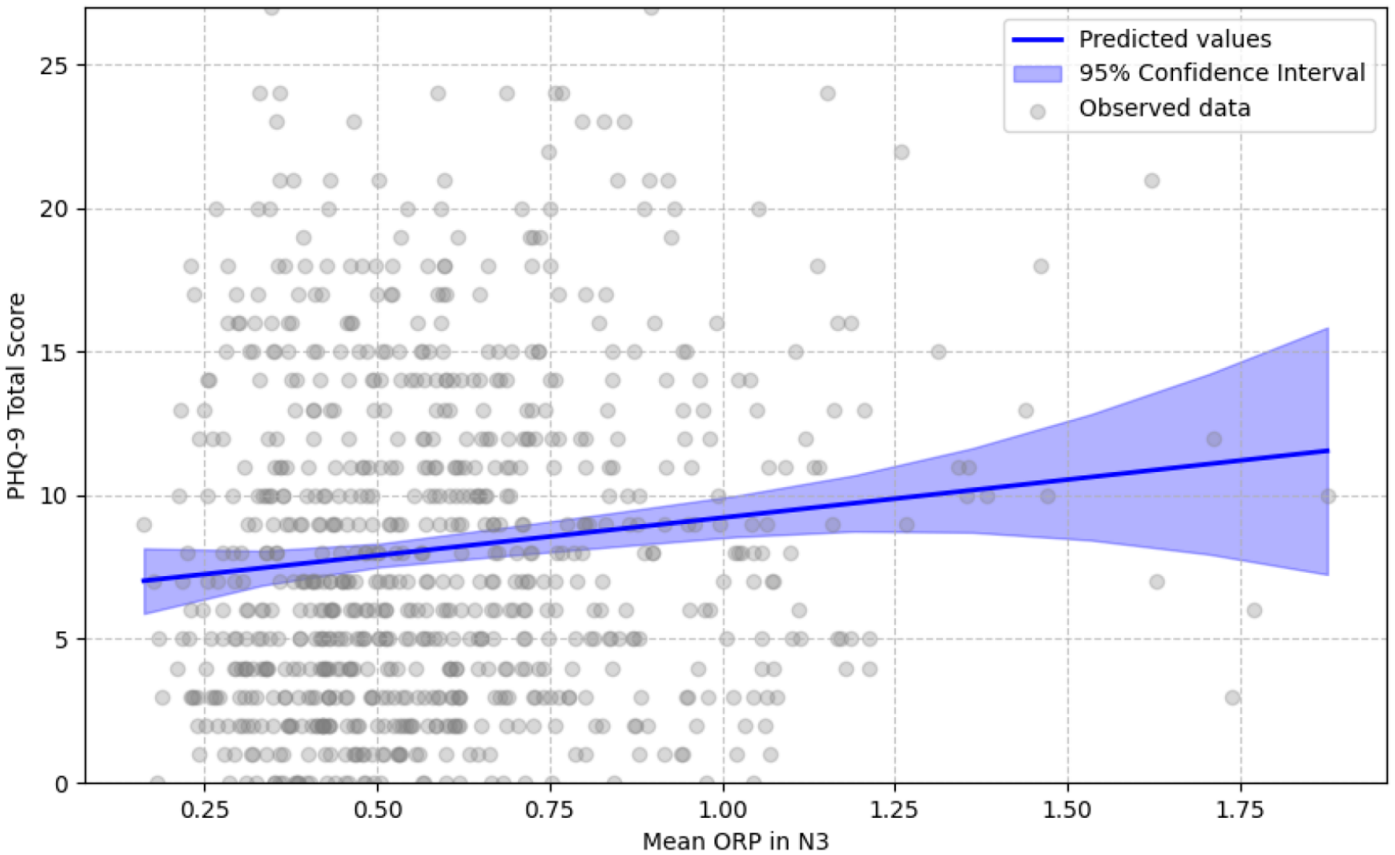
Linear relationship of mean ORP in N3 with PHQ-9 score. Gray circles show each participant's mean ORP during stage N3 (0 = very deep sleep, 2.5 = full wakefulness) against their total PHQ-9 score. The solid blue line represents values predicted by the best-fitting age- and sex-adjusted fixed-effects quadratic regression; the translucent band is the 95% confidence interval. The linear term reached statistical significance, while the quadratic term did not; consequently, the fitted relationship is strictly linear. This indicates that higher (shallower sleep) mean ORP values are associated with higher depressive-symptom burden.

When considering specific sleep stages, mean ORP during N1 + N2 also exhibited a non-significant linear relationship (β = 0.016, *p* = 0.625), but a positive significant quadratic effect was present (β_quadratic_ = 0.073, *p* = 0.003) that indicates a similar U-shaped relationship as observed above. Instead, the mean ORP in N3 showed a significant positive linear association with PHQ-9 scores (β = 0.12, *p* = 0.004), but no significant quadratic relationship (β_quadratic_ = 0.0, *p* = 0.991), signifying that as the mean ORP in N3 increases indicating shallower sleep in N3, individuals are expected to have higher PHQ-9 total scores (Figure 3). These findings suggest that ORP levels in N3 sleep might follow a more straightforward linear association with depression severity, whereas the relationship follows a quadratic trajectory for N1 + N2 and overall sleep stages. Finally, no significant association resulted for the mean ORP during the REM stage and during Wake epochs with the PHQ-9 total score.

All significant associations in the linear regression models could be interpreted as of small magnitude based on the conventional effect size interpretations that consider a β of 0.1 as indicative of a small effect (Cohen, 1988).

For the logistic regression models, in which a dichotomized PHQ-9 total score as above or below the threshold of 10 was used as the dependent variable, mean ORP during the whole PSG did not exhibit a significant linear relationship with increased depression risk (β = 0.042, *p* = 0.589), though a significant positive quadratic association was observed (β_quadratic_ = 0.248, *p* = 0.001), indicating a potential U-shaped relationship. This indicates that individuals with either higher or lower mean ORP values are at a higher risk of having a PHQ-9 total score above the threshold of 10 than individuals with average mean ORP values during the whole PSG.

Similarly, for N1+N2, the linear effect was non-significant (β = 0.02, *p* = 0.793), whereas a significant positive quadratic effect was found (βquadratic = 0.233, *p* = 0.002) that indicates a U-shaped relationship as observed above. In contrast, mean ORP in N3 showed a significant linear association with higher odds of having PHQ-9 scores above 10 (β = 0.255, *p* = 0.020), but the quadratic term was not significant (βquadratic = 0.041, *p* = 0.668).

All significant associations in the logistic regression models could be interpreted as having a small magnitude based on conventional standards, as a β below 0.3 is usually indicative of a small effect (Cohen, 1988).

These results highlight a complex relationship between mean ORP and PHQ-9. While ORP in deep sleep (N3) shows a more direct association with depression scores, ORP in lighter sleep stages (N1 + N2) and during the whole PSG exhibits non-linear, U-shaped patterns. Complete results are reported in Tables 2, 3.

**Table 2:** Association between ORP average values during overall and specific sleep stages and PHQ-9.

**PHQ-9 total score (Linear model)**		**Linear term**			**Quadratic term**				
	***N***	***b***	β	***p*** **-value (t)**	***b***	β	***p*** **-value (t)**	*R* ^2^	*R*^2^ **increase**
*avg ORP all stages*	829	0.400	0.022	0.521	4.6241	0.078	**0.001** ^*^	12.2%	1.2%
*avg ORP N1 + N2*	829	0.318	0.017	0.625	4.7361	0.073	**0.003** ^*^	12.1%	1.1%
*avg ORP N3*	790	2.623	0.120	**0.004** ^*^	0.0191	0.000	0.991	13.6%	1.4%
*avg ORP REM*	783	0.203	0.013	0.714	2.2185	0.049	0.082	13.1%	0.3%
*avg ORP wake*	829	0.447	0.014	0.75	2.9756	0.017	0.458	11.1%	0.1%
**PHQ-9** >= **10 (Logistic model)**		**Linear term**			**Quadratic term**				
	***N***	***b***	β	***p*** **-value (t)**	**b**	β	***p*** **-value (t)**	**AUROC**	**AUROC increase**
*avg ORP all stages*	829	0.134	0.042	0.589	1.879	0.248	**0.001** ^*^	0.681	0.019
*avg ORP N1+N2*	829	0.067	0.02	0.793	1.94	0.233	**0.002** ^*^	0.678	0.017
*avg ORP N3*	790	0.856	0.225	**0.023** ^*^	0.291	0.041	0.668	0.688	0.015
*avg ORP REM*	783	0.058	0.021	0.795	0.623	0.094	0.226	0.676	0.001
*avg ORP wake*	829	−0.048	−0.009	0.932	1.188	0.071	0.448	0.664	0.003

avg, average; AUROC, Area Under the Receiver Operating Characteristic Curve; b, unstandardized linear regression coefficient; *N*, number of subjects included in the analysis; N1, Non-REM Sleep Stage 1; N2, Non-REM Sleep Stage 2; N3, Non-REM Sleep Stage 3; OR, Odds-Ratio; ORP, Odds Ratio Product; PHQ-9, Patient Health Questionnaire, 9 items (major depression module); REM, Rapid Eye Movement; t, t-test; β, standardized linear regression coefficient; ^*^p < 0.05 (bolded values are significant).

**Table 3:** Association between ORP standard deviation values during overall and specific sleep stages and PHQ-9.

**PHQ-9 total score (Linear model)**		**Linear term**			**Quadratic term**				
	***N***	***b***	β	***p*** **-value (t)**	***b***	β	***p*** **-value (t)**	*R* ^2^	*R*^2^ **increase**
*sd ORP all stages*	829	−5.072	−0.104	**0.002** ^*^	−7.781	−0.019	0.422	12.0%	1.0%
*sd ORP N1 + N2*	829	−2.202	−0.031	0.38	−18.718	−0.022	0.393	11.2%	0.2%
*sd ORP N3*	790	7.720	0.095	**0.02** ^*^	−1.232	−0.001	0.949	13.0%	0.8%
*sd ORP REM*	783	−1.525	−0.018	0.59	3.017	0.002	0.897	12.8%	< 0.1%
*sd ORP wake*	829	0.582	0.008	0.837	51.268	0.053	**0.023** ^*^	11.8%	0.6%
**PHQ-9** >= **10 (Logistic model)**		**Linear term**			**Quadratic term**				
	***N***	***b***	β	***p*** **-value (t)**	***b***	β	***p*** **-value (t)**	**AUROC**	**AUROC increase**
*sd ORP all stages*	829	−1.929	−0.227	**0.005** ^*^	−3.925	−0.077	0.333	0.674	0.013
*sd ORP N1 + N2*	829	−0.234	−0.019	0.815	0.872	0.008	0.931	0.662	< 0.001
*sd ORP N3*	790	2.172	0.153	0.117	6.134	0.073	0.465	0.680	0.007
*sd ORP REM*	783	−0.334	−0.023	0.775	0.563	0.005	0.955	0.675	< 0.001
*sd ORP wake*	829	0.391	0.03	0.73	16.93	0.16	0.062	0.668	0.007

AUROC, Area Under the Receiver Operating Characteristic Curve; b, unstandardized linear regression coefficient; *N*, number of subjects included in the analysis; N1, Non-REM Sleep Stage 1; N2, Non-REM Sleep Stage 2; N3, Non-REM Sleep Stage 3; OR, Odds-Ratio; ORP, Odds Ratio Product; sd, standard deviation; PHQ-9, Patient Health Questionnaire, 9 items (major depression screening module); REM, Rapid Eye Movement; t, t-test; β, standardized linear regression coefficient; ^*^p < 0.05 (bolded values are significant).

### 3.5 Association of PHQ-9 with standard deviation ORP

For the standard deviation of ORP, the results were more nuanced. For the linear regression models, the standard deviation of ORP during the whole PSG exhibited a significant negative linear association with PHQ-9 total score (β = −0.104, *p* = 0.002), indicating that lower variability in ORP during the whole PSG was associated with higher depression severity. However, no significant quadratic relationship was found (β_quadratic_ = −0.019, *p* = 0.422). When examining specific sleep stages, the standard deviation of ORP in N1 + N2 showed no significant linear (β = −0.031, *p* = 0.380) or quadratic (β_quadratic_ = −0.022, *p* = 0.393) association with PHQ-9 total score. In contrast, the standard deviation of ORP in N3 displayed a significant positive linear association with PHQ-9 total score (*b* = 0.095, *p* = 0.020), indicating that lower variability in ORP during N3 was associated with lower depression. No quadratic relationship was found (β_quadratic_ = −0.001, *p* = 0.949). Finally, no significant association resulted for the standard deviation of ORP during the REM stage and during Wake epochs with PHQ-9 total score.

As for mean ORP, all significant associations in the linear regression models for the standard deviation of ORP could be interpreted as of small magnitude based on the conventional effect size interpretations that consider a b of 0.1 as indicative of a small effect (Cohen, 1988).

For the logistic regression models, a significant negative linear association of small magnitude (Cohen, 1988) was observed with the standard deviation of ORP during the whole PSG (β = −0.227, *p* = 0.005), indicating that higher variability in ORP was associated with a lower likelihood of depression scores above 10. However, no quadratic association was found (β_quadratic_ = −0.077, *p* = 0.333). For N1 + N2, N3, REM, and Wake, no significant associations were found for either the linear or quadratic terms.

These findings underscore a nuanced association between the variability of ORP and depression. Specifically, the standard deviation of ORP during the whole PSG was significantly and negatively associated with PHQ-9 scores in linear regression models, suggesting that greater overall variability is linked to lower depression severity, albeit with a small effect size. In contrast, variability during deep sleep (N3) showed a trend toward a positive association with depression, while no significant associations emerged in lighter sleep stages (N1 + N2), REM, or Wake periods, and no quadratic effects were observed in any model. Consistent with these findings, logistic regression revealed a significant negative linear association during the whole PSG. Complete results are reported in Tables 2, 3.

## 4 Discussion

Our study aimed to investigate whether and how depressive symptom severity is associated with the Odds Ratio Product (ORP), a sensitive EEG-derived index of sleep depth and arousability, across different sleep stages. We examined this relationship in a large cohort of individuals referred for sleep-wake disorder evaluations at multiple sleep centers (SCs) across the United States. This work addresses a critical gap, as no prior studies have assessed ORP in relation to depressive symptoms, despite known challenges in detecting non-linear or heterogeneous sleep alterations in depression using conventional PSG metrics.

Our primary finding was a significant quadratic (U-shaped) association between PHQ-9 scores and mean ORP across the full-night PSG (Figure 2). Both high and low ORP values were linked to elevated depression scores, suggesting that individuals with depressive symptoms may exhibit either hyperarousal (high ORP) or hypersomnolence (low ORP) phenotypes. This is consistent with the well-documented heterogeneity of sleep disturbances in depression, which can include both insomnia-like symptoms (e.g., increased fragmentation, prolonged sleep latency) and hypersomnia (e.g., increased total sleep time, excessive sleep depth) depending on subtype and individual variability (Geoffroy et al., 2018; Selvi et al., 2018).

Stage-specific analysis further supported this interpretation. A similar U-shaped relationship emerged between PHQ-9 scores and ORP in N1 + N2, while a positive linear association was observed in N3 (Figure 3), indicating that lighter deep sleep (higher ORP in N3) was associated with more severe depressive symptoms. These results align with evidence that depression is frequently accompanied by reduced slow-wave sleep (SWS) and diminished slow-wave activity (SWA), contributing to poor sleep quality, memory deficits, and emotional dysregulation (Benca et al., 1992; Armitage et al., 1992; Plante et al., 2012). No significant associations were found in REM or wake epochs. The lack of ORP correlation with REM may be attributable to unmeasured pharmacologic effects, particularly antidepressants, which are known to suppress REM duration and prolong REM onset latency (Wilson and Argyropoulos, 2005), though medication data were not available in this cohort.

We also observed a small but statistically significant negative linear relationship between the standard deviation of ORP over the entire PSG and PHQ-9 scores (Cohen, 1988), indicating that lower ORP variability may be associated with greater depressive symptom severity. This effect was most evident in N3 sleep. In contrast, no significant variability-related findings were observed in N1 + N2, REM, or wake stages. Reduced variability could reflect impaired sleep-state transitions or diminished neurophysiological flexibility in individuals with depression.

Our models adjusted for key demographic covariates. Age was negatively associated with PHQ-9 scores, consistent with epidemiological evidence of decreasing symptom severity with age. Female participants reported significantly higher depressive symptoms than males, reflecting well-established gender disparities in depression prevalence and onset, particularly emerging around puberty (Rahman et al., 2015).

Although the dataset included participants from multiple SCs, no meaningful center-level differences were observed in the association between ORP and depressive symptoms, supporting the generalizability of our findings across varied clinical environments. Nevertheless, future research should continue to examine whether institutional protocols, regional practices, or demographic differences contribute to site-specific variation.

In sum, our findings highlight ORP's potential as a sensitive, objective marker of depressive symptomatology, capable of capturing both hyperarousal and hypersomnolence within sleep architecture. This may inform the development of automated, physiology-based screening tools that passively detect depressive risk during standard PSG, addressing persistent gaps in depression recognition in sleep clinic settings.

## 5 Limitations

This study presents several limitations that should be considered when interpreting the findings. First, the retrospective design and reliance on data from two distinct studies may introduce heterogeneity due to variations in recruitment periods, clinical settings, and polysomnographic equipment, potentially affecting the results. However, this approach provides a more naturalistic picture, enhancing the generalizability of the findings. Second, while the PHQ-9 is a validated tool for assessing depressive symptoms, its use as a self-report measure is subject to biases and may not capture the full spectrum of depressive disorders, particularly in populations with comorbid conditions. This could lead to less reliable correspondence between subjective and objective measures. Third, the automated sleep staging and ORP computations, though efficient, depend on EEG signal quality; epochs with artifacts or signal loss were excluded, which might have led to data attrition and potential bias. Furthermore, the inclusion of participants on various psychotropic medications, without monitoring due to unavailable data, introduces potential confounding effects, as these medications can influence both sleep architecture and depressive symptomatology. Lastly, while mixed-effects models were employed to account for center-related variability, unmeasured confounding factors inherent to each site may still influence the results. Moreover, although our analyses were adjusted for the key demographic confounders of age and sex, we acknowledge that other potential influences, such as body mass index, comorbid sleep-disordered breathing, education, or the use of psychotropic and hypnotic medications, were not fully accounted for. Therefore, some residual confounding cannot be entirely dismissed in this initial study. These limitations will be addressed in future research.

## 6 Conclusion

Our findings highlight the potential utility of ORP analysis in clinical sleep settings as a non-invasive marker for depression severity. Given that ORP can be derived directly from standard PSG data, its integration into routine sleep assessments may enhance the identification of clinically significant depressive symptoms. Moreover, the observed patterns of ORP variability open promising avenues for developing machine learning models capable of screening for depressive burden based on sleep architecture and EEG-derived metrics. This approach could facilitate more personalized and efficient diagnostic pathways within sleep clinics, especially in populations presenting with sleep-wake disturbances.

## Data Availability

The datasets presented in this article are not readily available because the dataset is Medibio Limited property. Requests to access the datasets should be directed to massimiliano.grassi@medibio.com.au.
