# Supplementary material for: Clinical association between current depressive symptoms and odds ratio product in US sleep centers
Source: Front Sleep. 2025 Sep 12;4:1635704. doi: 10.3389/frsle.2025.1635704 (PMC12713867; doi:10.3389/frsle.2025.1635704)
Supplement: Supplementary file 1 [file Table_1.docx]

Table S1: PSG Equipment Used Per Site

| **SITE** | **SADB Study** | **SAMDE Phase 1**  **Study** | **SAMDE Phase 2 Study** | **PSG SYSTEM USED AT SITE** |
| --- | --- | --- | --- | --- |
| FL | No | Yes | No | Philips Respironics Sleepware G3 |
| MN | Yes | Yes | Yes | Somnostar v10.2 |
| NC | No | Yes | Yes | Philips Respironics Sleepware G3 |
| OH | Yes | Yes | Yes | Natus Neurology Sandman Elite v 10.1 |
| SC | No | Yes | Yes | Philips Respironics Sleepware G3 |
| TA | No | No | Yes | Natus Embla Rembrandt Analysis Manager |
| TX | No | No | Yes | Compumedics Profusion PSG 4 v 4.5 |

Table S2: Descriptive Statistics Per Site

| Site: | **MN (n=262)** | | **SC (n=202)** | | | **TX (n=121)** | | | **NC (n=113)** | | | **OH (n=107)** | | | **TA (n=14)** | | | **FL (n=10)** | | |  |
| --- | --- | --- | --- | --- | --- | --- | --- | --- | --- | --- | --- | --- | --- | --- | --- | --- | --- | --- | --- | --- | --- |
| Property | **Mean** | **S.D.** | | **Mean** | **S.D.** | | **Mean** | **S.D.** | | **Mean** | **S.D.** | | **Mean** | **S.D.** | | **Mean** | **S.D.** | | **Mean** | **S.D.** | |
| Age | 48.06 | 15.13 | | 37.90 | 11.12 | | 42.78 | 12.52 | | 45.11 | 12.22 | | 40.66 | 13.11 | | 48.64 | 13.80 | | 50.50 | 12.90 | |
| BMI | 33.29 | 9.40 | | 31.35 | 7.41 | | 27.93 | 7.37 | | 34.28 | 7.23 | | 35.99 | 10.82 | | 26.47 | 4.46 | | 33.29 | 9.76 | |
| Total Sleep Time (hours) | 6.08 | 1.02 | | 5.08 | 1.28 | | 5.53 | 1.21 | | 5.17 | 0.98 | | 4.93 | 1.04 | | 5.34 | 0.90 | | 5.85 | 0.46 | |
| Apnea-Hypopnea Index | 16.27 | 23.51 | | 10.85 | 15.13 | | 24.90 | 20.21 | | 15.91 | 20.57 | | 12.62 | 20.01 | | 20.74 | 12.42 | | 5.89 | 7.59 | |
| Sleep Efficiency | 83.58 | 11.76 | | 76.84 | 17.99 | | 78.99 | 13.56 | | 77.82 | 12.56 | | 74.50 | 14.34 | | 78.21 | 10.13 | | 85.46 | 6.55 | |
| SpO2 < 88% (percent) | 8.10% | 16.41% | | 1.57% | 4.13% | | 1.38% | 5.30% | | 3.60% | 7.72% | | 4.17% | 11.89% | | 3.99% | 5.12% | | 0.42% | 0.74% | |
| Respiratory Arousal Index | 13.78 | 20.68 | | 4.48 | 5.67 | | 23.18 | 15.19 | | 5.02 | 9.13 | | 14.33 | 14.80 | | 18.21 | 12.81 | | 2.70 | 2.89 | |
| Periodic Leg Movement Arousal Index | 0.92 | 2.00 | | 1.44 | 3.78 | | 0.54 | 2.45 | | 2.19 | 4.31 | | 1.92 | 4.43 | | 0.82 | 0.84 | | 1.78 | 2.22 | |
| Spontaneous Arousal Index | 8.90 | 5.50 | | 6.60 | 5.14 | | 2.07 | 3.46 | | 8.89 | 4.74 | | 12.90 | 6.99 | | 8.34 | 6.35 | | 8.38 | 5.10 | |
| Total Arousal Index | 23.75 | 20.53 | | 13.14 | 9.88 | | 25.84 | 15.67 | | 16.12 | 11.87 | | 28.91 | 16.93 | | 32.02 | 15.82 | | 15.78 | 6.42 | |
| PHQ-9 Total Score | 7.61 | 5.80 | | 8.48 | 5.28 | | 9.00 | 6.47 | | 7.13 | 5.15 | | 9.10 | 5.78 | | 7.79 | 6.60 | | 9.00 | 7.27 | |
| avg ORP all stages | 1.16 | 0.30 | | 1.41 | 0.27 | | 1.16 | 0.31 | | 1.32 | 0.26 | | 1.32 | 0.31 | | 1.44 | 0.37 | | 1.33 | 0.31 | |
| avg ORP N1+N2 | 1.01 | 0.27 | | 1.27 | 0.25 | | 0.98 | 0.28 | | 1.15 | 0.26 | | 1.19 | 0.31 | | 1.32 | 0.39 | | 1.26 | 0.31 | |
| avg ORP N3 | 0.46 | 0.20 | | 0.73 | 0.22 | | 0.52 | 0.23 | | 0.61 | 0.21 | | 0.70 | 0.31 | | 0.86 | 0.47 | | 0.71 | 0.29 | |
| avg ORP rem | 1.31 | 0.34 | | 1.64 | 0.28 | | 1.19 | 0.33 | | 1.50 | 0.27 | | 1.35 | 0.36 | | 1.61 | 0.36 | | 1.44 | 0.43 | |
| avg ORP wake | 2.19 | 0.17 | | 2.15 | 0.18 | | 2.15 | 0.18 | | 2.19 | 0.17 | | 2.11 | 0.19 | | 2.24 | 0.16 | | 2.28 | 0.10 | |
| std dev ORP all stages | 0.60 | 0.11 | | 0.50 | 0.11 | | 0.61 | 0.10 | | 0.58 | 0.12 | | 0.55 | 0.10 | | 0.57 | 0.14 | | 0.57 | 0.10 | |
| std dev ORP N1+N2 | 0.43 | 0.08 | | 0.35 | 0.07 | | 0.40 | 0.06 | | 0.40 | 0.08 | | 0.40 | 0.07 | | 0.44 | 0.10 | | 0.44 | 0.07 | |
| std dev ORP N3 | 0.19 | 0.07 | | 0.19 | 0.06 | | 0.22 | 0.08 | | 0.21 | 0.05 | | 0.22 | 0.07 | | 0.29 | 0.10 | | 0.23 | 0.07 | |
| std dev ORP rem | 0.29 | 0.06 | | 0.21 | 0.06 | | 0.30 | 0.06 | | 0.27 | 0.06 | | 0.27 | 0.07 | | 0.27 | 0.05 | | 0.29 | 0.06 | |
| std dev ORP wake | 0.20 | 0.07 | | 0.20 | 0.07 | | 0.22 | 0.08 | | 0.20 | 0.07 | | 0.22 | 0.09 | | 0.19 | 0.10 | | 0.15 | 0.06 | |

Table S3: Descriptive Statistics for Gender and BMI Per Site

| Site: | | **MN (n=262)** | | **SC (n=202)** | | **TX (n=121)** | | **NC (n=113)** | | **OH (n=107)** | | **TA (n=14)** | | **FL (n=10)** | |
| --- | --- | --- | --- | --- | --- | --- | --- | --- | --- | --- | --- | --- | --- | --- | --- |
|  |  | **N** | **%** | **N** | **%** | **N** | **%** | **N** | **%** | **N** | **%** | **N** | **%** | **N** | **%** |
| Gender | Male | 144 | 54.96% | 74 | 36.63% | 74 | 61.16% | 41 | 36.28% | 57 | 53.27% | 9 | 64.29% | 6 | 60.00% |
|  | Female | 118 | 45.04% | 128 | 63.37% | 47 | 38.84% | 72 | 63.72% | 50 | 46.73% | 5 | 35.71% | 4 | 40.00% |
| Body Mass Index | Underweight and Healthy Weight (BMI of 24.9 or less) | 52 | 19.85% | 25 | 12.38% | 48 | 39.67% | 6 | 5.31% | 10 | 9.35% | 6 | 42.86% | 2 | 20.00% |
|  | Overweight (BMI of 25-29.9) | 61 | 23.28% | 75 | 37.13% | 41 | 33.88% | 29 | 25.66% | 18 | 16.82% | 4 | 28.57% | 4 | 40.00% |
|  | Obesity (BMI of 30-39.9) | 97 | 37.02% | 82 | 40.59% | 24 | 19.83% | 54 | 47.79% | 52 | 48.60% | 4 | 28.57% | 2 | 20.00% |
|  | Severe Obesity (BMI of 40 or more) | 52 | 19.85% | 20 | 9.90% | 8 | 6.61% | 24 | 21.24% | 27 | 25.23% | - | - | 2 | 20.00% |

*Table S4: Detailed results* *of linear regression models with PHQ-9 total score as dependent variable and unstandardized independent variables*

| **ORP Variables** | **N** | **Model** | | | |  |  |
| --- | --- | --- | --- | --- | --- | --- | --- |
|  |  | **R-squared** | **Adjusted**  **R-squared** | **F** | **p-value (F)** |  |  |
| avg orp all stages | 829 | 0.122 | 0.118 | 28.697 | <.001* |  |  |
| avg orp N1+N2 | 829 | 0.121 | 0.117 | 28.308 | <.001* |  |  |
| avg orp N3 | 790 | 0.136 | 0.131 | 30.816 | <.001* |  |  |
| avg orp rem | 783 | 0.131 | 0.127 | 29.376 | <.001* |  |  |
| avg orp wake | 829 | 0.111 | 0.106 | 25.635 | <.001* |  |  |
| std dev all stages | 829 | 0.120 | 0.116 | 28.114 | <.001* |  |  |
| std dev N1+N2 | 829 | 0.112 | 0.107 | 25.902 | <.001* |  |  |
| std dev N3 | 790 | 0.130 | 0.125 | 29.253 | <.001* |  |  |
| std dev rem | 783 | 0.128 | 0.124 | 28.570 | <.001* |  |  |
| std dev wake | 829 | 0.118 | 0.113 | 27.496 | <.001* |  |  |
| N = sample size used in the analysis; R-squared = Coefficient of determination of the model; F = F-test of the coefficient of determination of the mod | | | | | | | |
|  | | | | | | | |
| **ORP Variables** | **N** | **Intercept** | | | | | |
|  |  | **Intercept** | **SE (intercept)** | **t** | **p-value** | **95% CI**  **(Lower Bound)** | **95% CI**  **(Higher Bound)** |
| avg orp all stages | 829 | 7.7237 | 0.233 | 33.124 | <.001* | 7.266 | 8.181 |
| avg orp N1+N2 | 829 | 7.752 | 0.235 | 32.966 | <.001* | 7.29 | 8.214 |
| avg orp N3 | 790 | 8.1594 | 0.223 | 36.58 | <.001* | 7.722 | 8.597 |
| avg orp rem | 783 | 7.8209 | 0.249 | 31.404 | <.001* | 7.332 | 8.31 |
| avg orp wake | 829 | 8.0754 | 0.229 | 35.297 | <.001* | 7.626 | 8.524 |
| std dev all stages | 829 | 8.279 | 0.231 | 35.882 | <.001* | 7.826 | 8.732 |
| std dev N1+N2 | 829 | 8.2956 | 0.238 | 34.842 | <.001* | 7.828 | 8.763 |
| std dev N3 | 790 | 8.1668 | 0.213 | 38.325 | <.001* | 7.749 | 8.585 |
| std dev rem | 783 | 8.087 | 0.219 | 36.987 | <.001* | 7.658 | 8.516 |
| std dev wake | 829 | 7.8654 | 0.231 | 34.085 | <.001* | 7.412 | 8.318 |
| N = sample size used in the analysis; b = unstandardized linear regression coefficient; SE (b) = standard error of the unstandardized linear regression coefficient; t = t-test of the intercept or of the regression coefficient; CI = confidence intervals * = p<0.05 | | | | | | | |
|  | | | | | | | |
| **ORP Variables** | **N** | **ORP Variable** | | | | | |
|  |  | **b** | **SE (b)** | **t** | **p-value (t)** | **95% CI**  **(Lower Bound)** | **95% CI**  **(Higher Bound)** |
| avg orp all stages | 829 | 0.3999 | 0.623 | 0.642 | 0.521 | -0.823 | 1.623 |
| avg orp N1+N2 | 829 | 0.3178 | 0.65 | 0.489 | 0.625 | -0.959 | 1.594 |
| avg orp N3 | 790 | 2.6226 | 0.912 | 2.875 | 0.004* | 0.832 | 4.413 |
| avg orp rem | 783 | 0.2027 | 0.553 | 0.366 | 0.714 | -0.884 | 1.289 |
| avg orp wake | 829 | 0.4472 | 1.406 | 0.318 | 0.75 | -2.312 | 3.206 |
| std dev all stages | 829 | -5.0724 | 1.669 | -3.04 | 0.002* | -8.348 | -1.797 |
| std dev N1+N2 | 829 | -2.2022 | 2.506 | -0.879 | 0.38 | -7.12 | 2.716 |
| std dev N3 | 790 | 7.72 | 3.299 | 2.34 | 0.02* | 1.244 | 14.196 |
| std dev rem | 783 | -1.5247 | 2.829 | -0.539 | 0.59 | -7.079 | 4.029 |
| std dev wake | 829 | 0.5824 | 2.828 | 0.206 | 0.837 | -4.969 | 6.133 |
| N = sample size used in the analysis; b = unstandardized linear regression coefficient; SE (b) = standard error of the unstandardized linear regression coefficient; t = t-test of the intercept or of the regression coefficient; CI = confidence intervals * = p<0.05 | | | | | | | |
|  | | | | | | | |
| **ORP Variables** | **N** | **ORP Variable (Quadratic Term)** | | | | | |
|  |  | **b** | **SE (b)** | **t** | **p-value (t)** | **95% CI**  **(Lower Bound)** | **95% CI**  **(Higher Bound)** |
| avg orp all stages | 829 | 4.6241 | 1.432 | 3.228 | 0.001* | 1.812 | 7.436 |
| avg orp N1+N2 | 829 | 4.7361 | 1.601 | 2.958 | 0.003* | 1.594 | 7.879 |
| avg orp N3 | 790 | 0.0191 | 1.707 | 0.011 | 0.991 | -3.332 | 3.37 |
| avg orp rem | 783 | 2.2185 | 1.273 | 1.743 | 0.082 | -0.28 | 4.717 |
| avg orp wake | 829 | 2.9756 | 4.012 | 0.742 | 0.458 | -4.899 | 10.851 |
| std dev all stages | 829 | -7.7818 | 9.692 | -0.803 | 0.422 | -26.806 | 11.242 |
| std dev N1+N2 | 829 | -18.7185 | 21.882 | -0.855 | 0.393 | -61.669 | 24.232 |
| std dev N3 | 790 | -1.232 | 19.391 | -0.064 | 0.949 | -39.296 | 36.832 |
| std dev rem | 783 | 3.017 | 23.282 | 0.13 | 0.897 | -42.686 | 48.72 |
| std dev wake | 829 | 51.268 | 22.434 | 2.285 | 0.023* | 7.234 | 95.302 |
| N = sample size used in the analysis; b = unstandardized linear regression coefficient; SE (b) = standard error of the unstandardized linear regression coefficient; t = t-test of the intercept or of the regression coefficient; CI = confidence intervals * = p<0.05 | | | | | | | |
|  | | | | | | | |
| **ORP Variables** | **N** | **Age** | | | | | |
|  |  | **b** | **SE (b)** | **t** | **p-value (t)** | **95% CI**  **(Lower Bound)** | **95% CI**  **(Higher Bound)** |
| avg orp all stages | 829 | -0.1319 | 0.014 | -9.407 | <.001* | -0.159 | -0.104 |
| avg orp N1+N2 | 829 | -0.13 | 0.014 | -9.434 | <.001* | -0.157 | -0.103 |
| avg orp N3 | 790 | -0.1337 | 0.014 | -9.512 | <.001* | -0.161 | -0.106 |
| avg orp rem | 783 | -0.1401 | 0.014 | -9.689 | <.001* | -0.168 | -0.112 |
| avg orp wake | 829 | -0.1289 | 0.014 | -8.901 | <.001* | -0.157 | -0.1 |
| std dev all stages | 829 | -0.1173 | 0.014 | -8.275 | <.001* | -0.145 | -0.089 |
| std dev N1+N2 | 829 | -0.1229 | 0.015 | -8.271 | <.001* | -0.152 | -0.094 |
| std dev N3 | 790 | -0.132 | 0.014 | -9.347 | <.001* | -0.16 | -0.104 |
| std dev rem | 783 | -0.1393 | 0.014 | -9.798 | <.001* | -0.167 | -0.111 |
| std dev wake | 829 | -0.1265 | 0.014 | -8.968 | <.001* | -0.154 | -0.099 |
| N = sample size used in the analysis; b = unstandardized linear regression coefficient; SE (b) = standard error of the unstandardized linear regression coefficient; t = t-test of the intercept or of the regression coefficient; CI = confidence intervals * = p<0.05 | | | | | | | |
|  | | | | | | | |
| **ORP Variables** | **N** | **Gender** | | | | | |
|  |  | **b** | **SE (b)** | **t** | **p-value (t)** | **95% CI**  **(Lower Bound)** | **95% CI**  **(Higher Bound)** |
| avg orp all stages | 829 | 1.5457 | -0.377 | 4.099 | <.001* | 2.286 | 0.806 |
| avg orp N1+N2 | 829 | 1.5601 | -0.376 | 4.146 | <.001* | 2.299 | 0.822 |
| avg orp N3 | 790 | 1.7918 | -0.382 | 4.691 | <.001* | 2.542 | 1.042 |
| avg orp rem | 783 | 1.6651 | -0.383 | 4.349 | <.001* | 2.417 | 0.914 |
| avg orp wake | 829 | 1.6241 | -0.383 | 4.24 | <.001* | 2.376 | 0.872 |
| std dev all stages | 829 | 1.6867 | -0.377 | 4.475 | <.001* | 2.427 | 0.947 |
| std dev N1+N2 | 829 | 1.6286 | -0.379 | 4.3 | <.001* | 2.372 | 0.885 |
| std dev N3 | 790 | 1.6348 | -0.386 | 4.238 | <.001* | 2.392 | 0.878 |
| std dev rem | 783 | 1.6907 | -0.385 | 4.396 | <.001* | 2.446 | 0.936 |
| std dev wake | 829 | 1.6525 | -0.379 | 4.363 | <.001* | 2.396 | 0.909 |
| N = sample size used in the analysis; b = unstandardized linear regression coefficient; SE (b) = standard error of the unstandardized linear regression coefficient; t = t-test of the intercept or of the regression coefficient; CI = confidence intervals * = p<0.05 | | | | | | | |

*Table S5: Detailed results of linear regression models with PHQ-9 total score as dependent variable and standardized independent variables*

| **ORP Variables** | **N** | **Model** | | | |  |  |
| --- | --- | --- | --- | --- | --- | --- | --- |
|  |  | **R-squared** | **Adjusted R-squared** | **F** | **p-value (F)** |  |  |
| avg orp all stages | 829 | 0.122 | 0.118 | 28.697 | <.001* |  |  |
| avg orp N1+N2 | 829 | 0.121 | 0.117 | 28.308 | <.001* |  |  |
| avg orp N3 | 790 | 0.136 | 0.131 | 30.816 | <.001* |  |  |
| avg orp rem | 783 | 0.131 | 0.127 | 29.376 | <.001* |  |  |
| avg orp wake | 829 | 0.111 | 0.106 | 25.635 | <.001* |  |  |
| std dev all stages | 829 | 0.120 | 0.116 | 28.114 | <.001* |  |  |
| std dev N1+N2 | 829 | 0.112 | 0.107 | 25.902 | <.001* |  |  |
| std dev N3 | 790 | 0.130 | 0.125 | 29.253 | <.001* |  |  |
| std dev rem | 783 | 0.128 | 0.124 | 28.570 | <.001* |  |  |
| std dev wake | 829 | 0.118 | 0.113 | 27.496 | <.001* |  |  |
| N = sample size used in the analysis; R-squared = Coefficient of determination of the model; F = F-test of the coefficient of determination of the mod | | | | | | | |
|  | | | | | | | |
| **ORP Variables** | **N** | **Intercept** | | | | | |
|  |  | **Intercept** | **SE (intercept)** | **t** | **p-value** | **95% CI**  **(Lower Bound)** | **95% CI**  **(Higher Bound)** |
| avg orp all stages | 829 | -0.0779 | 0.041 | -1.919 | 0.055 | -0.157 | 0.002 |
| avg orp N1+N2 | 829 | -0.0729 | 0.041 | -1.783 | 0.075 | -0.153 | 0.007 |
| avg orp N3 | 790 | -0.0002 | 0.039 | -0.006 | 0.995 | -0.077 | 0.076 |
| avg orp rem | 783 | -0.0491 | 0.044 | -1.124 | 0.261 | -0.135 | 0.037 |
| avg orp wake | 829 | -0.0167 | 0.04 | -0.419 | 0.675 | -0.095 | 0.061 |
| std dev all stages | 829 | 0.0187 | 0.04 | 0.467 | 0.641 | -0.06 | 0.098 |
| std dev N1+N2 | 829 | 0.0216 | 0.041 | 0.522 | 0.602 | -0.06 | 0.103 |
| std dev N3 | 790 | 0.0011 | 0.037 | 0.029 | 0.977 | -0.072 | 0.074 |
| std dev rem | 783 | -0.0024 | 0.038 | -0.063 | 0.949 | -0.078 | 0.073 |
| std dev wake | 829 | -0.0532 | 0.04 | -1.326 | 0.185 | -0.132 | 0.026 |
| N = sample size used in the analysis; β = standardized linear regression coefficient; SE (β) = standard error of the standardized linear regression coefficient.  t = t-test of the intercept or of the regression coefficient; CI = confidence intervals * = p<0.05 | | | | | | | |
|  | | | | | | | |
| **ORP Variables** | **N** | **ORP Variable** | | | | | |
|  |  | **β** | **SE (β)** | **t** | **p-value (t)** | **95% CI**  **(Lower Bound)** | **95% CI**  **(Higher Bound)** |
| avg orp all stages | 829 | 0.0217 | 0.034 | 0.642 | 0.521 | -0.045 | 0.088 |
| avg orp N1+N2 | 829 | 0.0165 | 0.034 | 0.489 | 0.625 | -0.05 | 0.083 |
| avg orp N3 | 790 | 0.1201 | 0.042 | 2.875 | 0.004* | 0.038 | 0.202 |
| avg orp rem | 783 | 0.0126 | 0.035 | 0.366 | 0.714 | -0.055 | 0.08 |
| avg orp wake | 829 | 0.014 | 0.044 | 0.318 | 0.75 | -0.072 | 0.1 |
| std dev all stages | 829 | -0.1039 | 0.034 | -3.04 | 0.002* | -0.171 | -0.037 |
| std dev N1+N2 | 829 | -0.0312 | 0.036 | -0.879 | 0.38 | -0.101 | 0.039 |
| std dev N3 | 790 | 0.0948 | 0.04 | 2.34 | 0.02* | 0.015 | 0.174 |
| std dev rem | 783 | -0.0181 | 0.034 | -0.539 | 0.59 | -0.084 | 0.048 |
| std dev wake | 829 | 0.0078 | 0.038 | 0.206 | 0.837 | -0.067 | 0.082 |
| N = sample size used in the analysis; β = standardized linear regression coefficient; SE (β) = standard error of the standardized linear regression coefficient.  t = t-test of the intercept or of the regression coefficient; CI = confidence intervals * = p<0.05 | | | | | | | |
|  | | | | | | | |
| **ORP Variables** | **N** | **ORP Variable (Quadratic Term)** | | | | | |
|  |  | **β** | **SE (β)** | **t** | **p-value (t)** | **95% CI**  **(Lower Bound)** | **95% CI**  **(Higher Bound)** |
| avg orp all stages | 829 | 0.078 | 0.024 | 3.228 | 0.001* | 0.031 | 0.125 |
| avg orp N1+N2 | 829 | 0.073 | 0.025 | 2.958 | 0.003* | 0.025 | 0.121 |
| avg orp N3 | 790 | 0.0002 | 0.021 | 0.011 | 0.991 | -0.04 | 0.04 |
| avg orp rem | 783 | 0.0492 | 0.028 | 1.743 | 0.082 | -0.006 | 0.105 |
| avg orp wake | 829 | 0.0167 | 0.023 | 0.742 | 0.458 | -0.028 | 0.061 |
| std dev all stages | 829 | -0.0188 | 0.023 | -0.803 | 0.422 | -0.065 | 0.027 |
| std dev N1+N2 | 829 | -0.0217 | 0.025 | -0.855 | 0.393 | -0.071 | 0.028 |
| std dev N3 | 790 | -0.0011 | 0.017 | -0.064 | 0.949 | -0.034 | 0.032 |
| std dev rem | 783 | 0.0024 | 0.019 | 0.13 | 0.897 | -0.034 | 0.039 |
| std dev wake | 829 | 0.0533 | 0.023 | 2.285 | 0.023* | 0.008 | 0.099 |
| N = sample size used in the analysis; β = standardized linear regression coefficient; SE (β) = standard error of the standardized linear regression coefficient.  t = t-test of the intercept or of the regression coefficient; CI = confidence intervals * = p<0.05 | | | | | | | |
|  | | | | | | | |
| **ORP Variables** | **N** | **Age** | | | | | |
|  |  | **β** | **SE (β)** | **t** | **p-value (t)** | **95% CI**  **(Lower Bound)** | **95% CI**  **(Higher Bound)** |
| avg orp all stages | 829 | -0.3153 | 0.034 | -9.407 | <.001* | -0.381 | -0.25 |
| avg orp N1+N2 | 829 | -0.3106 | 0.033 | -9.434 | <.001* | -0.375 | -0.246 |
| avg orp N3 | 790 | -0.3184 | 0.033 | -9.512 | <.001* | -0.384 | -0.253 |
| avg orp rem | 783 | -0.3333 | 0.034 | -9.689 | <.001* | -0.401 | -0.266 |
| avg orp wake | 829 | -0.308 | 0.035 | -8.901 | <.001* | -0.376 | -0.24 |
| std dev all stages | 829 | -0.2803 | 0.034 | -8.275 | <.001* | -0.347 | -0.214 |
| std dev N1+N2 | 829 | -0.2937 | 0.036 | -8.271 | <.001* | -0.363 | -0.224 |
| std dev N3 | 790 | -0.3143 | 0.034 | -9.347 | <.001* | -0.38 | -0.248 |
| std dev rem | 783 | -0.3314 | 0.034 | -9.798 | <.001* | -0.398 | -0.265 |
| std dev wake | 829 | -0.3024 | 0.034 | -8.968 | <.001* | -0.369 | -0.236 |
| N = sample size used in the analysis; β = standardized linear regression coefficient; SE (β) = standard error of the standardized linear regression coefficient;  t = t-test of the intercept or of the regression coefficient; CI = confidence intervals * = p<0.05 | | | | | | | |
|  | | | | | | | |
| **ORP Variables** | **N** | **Gender** | | | | | |
|  |  | **β** | **SE (β)** | **t** | **p-value (t)** | **95% CI**  **(Lower Bound)** | **95% CI**  **(Higher Bound)** |
| avg orp all stages | 829 | 0.1345 | -0.033 | 4.099 | <.001* | 0.199 | 0.07 |
| avg orp N1+N2 | 829 | 0.1358 | -0.033 | 4.146 | <.001* | 0.2 | 0.071 |
| avg orp N3 | 790 | 0.1566 | -0.033 | 4.691 | <.001* | 0.222 | 0.091 |
| avg orp rem | 783 | 0.1461 | -0.034 | 4.349 | <.001* | 0.212 | 0.08 |
| avg orp wake | 829 | 0.1413 | -0.033 | 4.24 | <.001* | 0.207 | 0.076 |
| std dev all stages | 829 | 0.1468 | -0.033 | 4.475 | <.001* | 0.211 | 0.082 |
| std dev N1+N2 | 829 | 0.1417 | -0.033 | 4.3 | <.001* | 0.206 | 0.077 |
| std dev N3 | 790 | 0.1429 | -0.034 | 4.238 | <.001* | 0.209 | 0.077 |
| std dev rem | 783 | 0.1483 | -0.034 | 4.396 | <.001* | 0.215 | 0.082 |
| std dev wake | 829 | 0.1438 | -0.033 | 4.363 | <.001* | 0.208 | 0.079 |
| N = sample size used in the analysis; β = standardized linear regression coefficient; SE (β) = standard error of the standardized linear regression coefficient.  t = t-test of the intercept or of the regression coefficient; CI = confidence intervals * = p<0.05 | | | | | | | |

*Table S6: Detailed results of logistic regression models with PHQ-9 cut-off >=10 as dependent variable and unstandardized independent variables*

| **ORP Variables** | **N** | **Model** | | |  |  |  |
| --- | --- | --- | --- | --- | --- | --- | --- |
|  |  | **Pseudo**  **R-squared** | **Log-Likelihood** | **p-value**  **(Chi-square)** |  |  |  |
| avg orp all stages | 829 | 0.071 | -506.428 | <.001* |  |  |  |
| avg orp N1+N2 | 829 | 0.070 | -507.122 | <.001* |  |  |  |
| avg orp N3 | 790 | 0.081 | -476.322 | <.001* |  |  |  |
| avg orp rem | 783 | 0.072 | -476.713 | <.001* |  |  |  |
| avg orp wake | 829 | 0.062 | -511.803 | <.001* |  |  |  |
| std dev all stages | 829 | 0.068 | -508.271 | <.001* |  |  |  |
| std dev N1+N2 | 829 | 0.061 | -512.326 | <.001* |  |  |  |
| std dev N3 | 790 | 0.077 | -478.381 | <.001* |  |  |  |
| std dev rem | 783 | 0.071 | -477.410 | <.001* |  |  |  |
| std dev wake | 829 | 0.066 | -509.497 | <.001* |  |  |  |
| N = sample size used in the analysis; Pseudo R-squared = Pseudo coefficient of determination of the model; | | | | | | | |
|  | | | | | | | |
| **ORP Variables** | **N** | **Intercept** | | | | | |
|  |  | **b** | **SE (b)** | **z** | **p-value (z)** | **95% CI**  **(Lower Bound)** | **95% CI**  **(Higher Bound)** |
| avg orp all stages | 829 | -0.782 | 0.096 | -8.127 | <.001* | -0.970 | -0.593 |
| avg orp N1+N2 | 829 | -0.772 | 0.097 | -7.974 | <.001* | -0.961 | -0.582 |
| avg orp N3 | 790 | -0.650 | 0.092 | -7.059 | <.001* | -0.830 | -0.469 |
| avg orp rem | 783 | -0.696 | 0.104 | -6.697 | <.001* | -0.900 | -0.492 |
| avg orp wake | 829 | -0.631 | 0.092 | -6.872 | <.001* | -0.811 | -0.451 |
| std dev all stages | 829 | -0.547 | 0.093 | -5.872 | <.001* | -0.729 | -0.364 |
| std dev N1+N2 | 829 | -0.598 | 0.096 | -6.252 | <.001* | -0.786 | -0.411 |
| std dev N3 | 790 | -0.651 | 0.089 | -7.345 | <.001* | -0.824 | -0.477 |
| std dev rem | 783 | -0.619 | 0.092 | -6.748 | <.001* | -0.798 | -0.439 |
| std dev wake | 829 | -0.696 | 0.094 | -7.384 | <.001* | -0.880 | -0.511 |
| N = sample size used in the analysis; b = unstandardized linear regression coefficient; SE (b) = standard error of the unstandardized linear regression coefficient; CI = confidence intervals; * = p<0.05; | | | | | | | |
|  | | | | | | | |
| **ORP Variables** | **N** | **ORP Variable** | | | | | |
|  |  | **b** | **SE (b)** | **z** | **p-value (z)** | **95% CI**  **(Lower Bound)** | **95% CI**  **(Higher Bound)** |
| avg orp all stages | 829 | 0.134 | 0.247 | 0.541 | 0.589 | -0.351 | 0.619 |
| avg orp N1+N2 | 829 | 0.067 | 0.257 | 0.262 | 0.793 | -0.436 | 0.570 |
| avg orp N3 | 790 | 0.856 | 0.376 | 2.279 | 0.023 | 0.120 | 1.593 |
| avg orp rem | 783 | 0.058 | 0.224 | 0.259 | 0.795 | -0.381 | 0.497 |
| avg orp wake | 829 | -0.048 | 0.564 | -0.086 | 0.932 | -1.153 | 1.056 |
| std dev all stages | 829 | -1.929 | 0.693 | -2.784 | 0.005 | -3.286 | -0.571 |
| std dev N1+N2 | 829 | -0.234 | 1.000 | -0.234 | 0.815 | -2.194 | 1.726 |
| std dev N3 | 790 | 2.172 | 1.384 | 1.569 | 0.117 | -0.541 | 4.885 |
| std dev rem | 783 | -0.334 | 1.169 | -0.286 | 0.775 | -2.626 | 1.958 |
| std dev wake | 829 | 0.391 | 1.132 | 0.345 | 0.730 | -1.828 | 2.610 |
| N = sample size used in the analysis; b = unstandardized linear regression coefficient; SE (b) = standard error of the unstandardized linear regression coefficient; CI = confidence intervals; * = p<0.05 | | | | | | | |
|  | | | | | | | |
| **ORP Variables** | **N** | **ORP Variable (Quadratic Term)** | | | | | |
|  |  | **b** | **SE (b)** | **z** | **p-value (z)** | **95% CI**  **(Lower Bound)** | **95% CI**  **(Higher Bound)** |
| avg orp all stages | 829 | 1.879 | 0.568 | 3.310 | 0.001 | 0.766 | 2.991 |
| avg orp N1+N2 | 829 | 1.940 | 0.631 | 3.074 | 0.002 | 0.703 | 3.176 |
| avg orp N3 | 790 | 0.291 | 0.679 | 0.428 | 0.668 | -1.039 | 1.621 |
| avg orp rem | 783 | 0.623 | 0.514 | 1.211 | 0.226 | -0.385 | 1.631 |
| avg orp wake | 829 | 1.188 | 1.566 | 0.759 | 0.448 | -1.881 | 4.258 |
| std dev all stages | 829 | -3.925 | 4.053 | -0.968 | 0.333 | -11.868 | 4.019 |
| std dev N1+N2 | 829 | 0.872 | 8.761 | 0.099 | 0.921 | -16.300 | 18.044 |
| std dev N3 | 790 | 6.133 | 8.400 | 0.730 | 0.465 | -10.330 | 22.597 |
| std dev rem | 783 | 0.563 | 9.908 | 0.057 | 0.955 | -18.858 | 19.983 |
| std dev wake | 829 | 16.930 | 9.069 | 1.867 | 0.062 | -0.845 | 34.705 |
| N = sample size used in the analysis; b = unstandardized linear regression coefficient; SE (b) = standard error of the unstandardized linear regression coefficient; CI = confidence intervals; * = p<0.05 | | | | | | | |
|  | | | | | | | |
| **ORP Variables** | **N** | **Age** | | | | | |
|  |  | **b** | **SE (b)** | **z** | **p-value (z)** | **95% CI**  **(Lower Bound)** | **95% CI**  **(Higher Bound)** |
| avg orp all stages | 829 | -0.043 | 0.006 | -7.111 | <.001* | -0.055 | -0.031 |
| avg orp N1+N2 | 829 | -0.043 | 0.006 | -7.120 | <.001* | -0.054 | -0.031 |
| avg orp N3 | 790 | -0.046 | 0.006 | -7.350 | <.001* | -0.059 | -0.034 |
| avg orp rem | 783 | -0.047 | 0.006 | -7.205 | <.001* | -0.059 | -0.034 |
| avg orp wake | 829 | -0.041 | 0.006 | -6.611 | <.001* | -0.053 | -0.029 |
| std dev all stages | 829 | -0.038 | 0.006 | -6.265 | <.001* | -0.049 | -0.026 |
| std dev N1+N2 | 829 | -0.041 | 0.006 | -6.525 | <.001* | -0.053 | -0.029 |
| std dev N3 | 790 | -0.045 | 0.006 | -7.229 | <.001* | -0.057 | -0.033 |
| std dev rem | 783 | -0.046 | 0.006 | -7.332 | <.001* | -0.059 | -0.034 |
| std dev wake | 829 | -0.041 | 0.006 | -6.741 | <.001* | -0.052 | -0.029 |
| N = sample size used in the analysis; b = unstandardized linear regression coefficient; SE (b) = standard error of the unstandardized linear regression coefficient; CI = confidence intervals; * = p<0.05 | | | | | | | |
|  | | | | | | | |
| **ORP Variables** | **N** | **Gender** | | | | | |
|  |  | **b** | **SE (b)** | **z** | **p-value (z)** | **95% CI**  **(Lower Bound)** | **95% CI**  **(Higher Bound)** |
| avg orp all stages | 829 | -0.540 | 0.152 | -3.547 | <.001* | -0.839 | -0.242 |
| avg orp N1+N2 | 829 | -0.544 | 0.152 | -3.585 | <.001* | -0.841 | -0.247 |
| avg orp N3 | 790 | -0.653 | 0.158 | -4.123 | <.001* | -0.963 | -0.343 |
| avg orp rem | 783 | -0.589 | 0.157 | -3.747 | <.001* | -0.897 | -0.281 |
| avg orp wake | 829 | -0.578 | 0.153 | -3.773 | <.001* | -0.878 | -0.278 |
| std dev all stages | 829 | -0.590 | 0.152 | -3.880 | <.001* | -0.888 | -0.292 |
| std dev N1+N2 | 829 | -0.562 | 0.151 | -3.715 | <.001* | -0.858 | -0.266 |
| std dev N3 | 790 | -0.596 | 0.158 | -3.772 | <.001* | -0.906 | -0.286 |
| std dev rem | 783 | -0.594 | 0.158 | -3.766 | <.001* | -0.903 | -0.285 |
| std dev wake | 829 | -0.582 | 0.152 | -3.814 | <.001* | -0.880 | -0.283 |
| N = sample size used in the analysis; b = unstandardized linear regression coefficient; SE (b) = standard error of the unstandardized linear regression coefficient; CI = confidence intervals; * = p<0.05 | | | | | | | |

*Table S7: Detailed results of logistic regression models with PHQ-9 cut-off >=10 as dependent variable and standardized independent variables*

| **ORP Variables** | **N** | **Model** | | |  |  |  |
| --- | --- | --- | --- | --- | --- | --- | --- |
|  |  | **Pseudo**  **R-squared** | **Log-Likelihood** | **p-value**  **(Chi-square)** |  |  |  |
| avg orp all stages | 829 | 0.071 | -506.428 | <.001 |  |  |  |
| avg orp N1+N2 | 829 | 0.070 | -507.122 | <.001 |  |  |  |
| avg orp N3 | 790 | 0.081 | -476.322 | <.001 |  |  |  |
| avg orp rem | 783 | 0.072 | -476.713 | <.001 |  |  |  |
| avg orp wake | 829 | 0.062 | -511.803 | <.001 |  |  |  |
| std dev all stages | 829 | 0.068 | -508.271 | <.001 |  |  |  |
| std dev N1+N2 | 829 | 0.061 | -512.326 | <.001 |  |  |  |
| std dev N3 | 790 | 0.077 | -478.381 | <.001 |  |  |  |
| std dev rem | 783 | 0.071 | -477.410 | <.001 |  |  |  |
| std dev wake | 829 | 0.066 | -509.497 | <.001 |  |  |  |
| N = sample size used in the analysis; Pseudo R-squared = Pseudo coefficient of determination of the model; | | | | | | | |
|  | | | | | | | |
| **ORP Variables** | **N** | **Intercept** | | | | | |
|  |  | **β** | **SE (β)** | **z** | **p-value (z)** | **95% CI**  **(Lower Bound)** | **95% CI**  **(Higher Bound)** |
| avg orp all stages | 829 | 7.724 | 0.233 | 33.124 | <.001 | 7.266 | 8.181 |
| avg orp N1+N2 | 829 | 7.752 | 0.235 | 32.966 | <.001 | 7.290 | 8.214 |
| avg orp N3 | 790 | 8.159 | 0.223 | 36.580 | <.001 | 7.722 | 8.597 |
| avg orp rem | 783 | 7.821 | 0.249 | 31.404 | <.001 | 7.332 | 8.310 |
| avg orp wake | 829 | 8.075 | 0.229 | 35.297 | <.001 | 7.626 | 8.524 |
| std dev all stages | 829 | 8.279 | 0.231 | 35.882 | <.001 | 7.826 | 8.732 |
| std dev N1+N2 | 829 | 8.296 | 0.238 | 34.842 | <.001 | 7.828 | 8.763 |
| std dev N3 | 790 | 8.167 | 0.213 | 38.325 | <.001 | 7.749 | 8.585 |
| std dev rem | 783 | 8.087 | 0.219 | 36.987 | <.001 | 7.658 | 8.516 |
| std dev wake | 829 | 7.865 | 0.231 | 34.085 | <.001 | 7.412 | 8.318 |
| N = sample size used in the analysis; β = standardized linear regression coefficient; SE (β) = standard error of the standardized linear regression coefficient; Z = Z-test of the intercept or of the regression coefficient; CI = confidence intervals; * = p<0.05. | | | | | | | |
|  | | | | | | | |
| **ORP Variables** | **N** | **ORP Variable** | | | | | |
|  |  | **β** | **SE (β)** | **z** | **p-value (z)** | **95% CI**  **(Lower Bound)** | **95% CI**  **(Higher Bound)** |
| avg orp all stages | 829 | 0.125 | 0.194 | 0.642 | 0.521 | -0.256 | 0.505 |
| avg orp N1+N2 | 829 | 0.095 | 0.194 | 0.489 | 0.625 | -0.285 | 0.475 |
| avg orp N3 | 790 | 0.688 | 0.239 | 2.875 | 0.004 | 0.218 | 1.157 |
| avg orp rem | 783 | 0.072 | 0.197 | 0.366 | 0.714 | -0.314 | 0.458 |
| avg orp wake | 829 | 0.080 | 0.253 | 0.318 | 0.750 | -0.415 | 0.576 |
| std dev all stages | 829 | -0.597 | 0.196 | -3.040 | 0.002 | -0.983 | -0.212 |
| std dev N1+N2 | 829 | -0.180 | 0.204 | -0.879 | 0.380 | -0.581 | 0.221 |
| std dev N3 | 790 | 0.542 | 0.232 | 2.340 | 0.020 | 0.087 | 0.997 |
| std dev rem | 783 | -0.103 | 0.192 | -0.539 | 0.590 | -0.480 | 0.273 |
| std dev wake | 829 | 0.045 | 0.219 | 0.206 | 0.837 | -0.384 | 0.474 |
| N = sample size used in the analysis; β = standardized linear regression coefficient; SE (β) = standard error of the standardized linear regression coefficient; Z = Z-test of the intercept or of the regression coefficient; CI = confidence intervals; * = p<0.05. | | | | | | | |
|  | | | | | | | |
| **ORP Variables** | **N** | **ORP Variable (Quadratic Term)** | | | | | |
|  |  | **β** | **SE (β)** | **z** | **p-value (z)** | **95% CI**  **(Lower Bound)** | **95% CI**  **(Higher Bound)** |
| avg orp all stages | 829 | 0.448 | 0.139 | 3.228 | 0.001 | 0.176 | 0.721 |
| avg orp N1+N2 | 829 | 0.420 | 0.142 | 2.958 | 0.003 | 0.141 | 0.698 |
| avg orp N3 | 790 | 0.001 | 0.117 | 0.011 | 0.991 | -0.229 | 0.232 |
| avg orp rem | 783 | 0.280 | 0.161 | 1.743 | 0.082 | -0.035 | 0.596 |
| avg orp wake | 829 | 0.096 | 0.129 | 0.742 | 0.458 | -0.158 | 0.350 |
| std dev all stages | 829 | -0.108 | 0.134 | -0.803 | 0.422 | -0.372 | 0.156 |
| std dev N1+N2 | 829 | -0.125 | 0.145 | -0.855 | 0.393 | -0.410 | 0.161 |
| std dev N3 | 790 | -0.006 | 0.096 | -0.064 | 0.949 | -0.194 | 0.182 |
| std dev rem | 783 | 0.014 | 0.107 | 0.130 | 0.897 | -0.196 | 0.224 |
| std dev wake | 829 | 0.306 | 0.134 | 2.285 | 0.023 | 0.043 | 0.569 |
| N = sample size used in the analysis; β = standardized linear regression coefficient; SE (β) = standard error of the standardized linear regression coefficient; Z = Z-test of the intercept or of the regression coefficient; CI = confidence intervals; * = p<0.05. | | | | | | | |
|  | | | | | | | |
| **ORP Variables** | **N** | **Age** | | | | | |
|  |  | **β** | **SE (β)** | **z** | **p-value (z)** | **95% CI**  **(Lower Bound)** | **95% CI**  **(Higher Bound)** |
| avg orp all stages | 829 | -1.812 | 0.193 | -9.407 | <.001 | -2.191 | -1.434 |
| avg orp N1+N2 | 829 | -1.785 | 0.189 | -9.434 | <.001 | -2.157 | -1.414 |
| avg orp N3 | 790 | -1.822 | 0.192 | -9.512 | <.001 | -2.198 | -1.446 |
| avg orp rem | 783 | -1.900 | 0.196 | -9.689 | <.001 | -2.285 | -1.515 |
| avg orp wake | 829 | -1.770 | 0.199 | -8.901 | <.001 | -2.161 | -1.380 |
| std dev all stages | 829 | -1.611 | 0.195 | -8.275 | <.001 | -1.993 | -1.229 |
| std dev N1+N2 | 829 | -1.688 | 0.204 | -8.271 | <.001 | -2.089 | -1.288 |
| std dev N3 | 790 | -1.799 | 0.192 | -9.347 | <.001 | -2.177 | -1.421 |
| std dev rem | 783 | -1.889 | 0.193 | -9.798 | <.001 | -2.267 | -1.511 |
| std dev wake | 829 | -1.738 | 0.194 | -8.968 | <.001 | -2.119 | -1.358 |
| N = sample size used in the analysis; β = standardized linear regression coefficient; SE (β) = standard error of the standardized linear regression coefficient; Z = Z-test of the intercept or of the regression coefficient; CI = confidence intervals; * = p<0.05. | | | | | | | |
|  | | | | | | | |
| **ORP Variables** | **N** | **Gender** | | | | | |
|  |  | **β** | **SE (β)** | **z** | **p-value (z)** | **95% CI**  **(Lower Bound)** | **95% CI**  **(Higher Bound)** |
| avg orp all stages | 829 | -0.773 | 0.189 | -4.099 | <.001 | -1.143 | -0.403 |
| avg orp N1+N2 | 829 | -0.780 | 0.188 | -4.146 | <.001 | -1.150 | -0.411 |
| avg orp N3 | 790 | -0.897 | 0.191 | -4.691 | <.001 | -1.272 | -0.521 |
| avg orp rem | 783 | -0.833 | 0.191 | -4.349 | <.001 | -1.209 | -0.457 |
| avg orp wake | 829 | -0.812 | 0.192 | -4.240 | <.001 | -1.188 | -0.436 |
| std dev all stages | 829 | -0.844 | 0.189 | -4.475 | <.001 | -1.214 | -0.474 |
| std dev N1+N2 | 829 | -0.815 | 0.189 | -4.300 | <.001 | -1.186 | -0.443 |
| std dev N3 | 790 | -0.818 | 0.193 | -4.238 | <.001 | -1.197 | -0.439 |
| std dev rem | 783 | -0.846 | 0.192 | -4.396 | <.001 | -1.223 | -0.468 |
| std dev wake | 829 | -0.827 | 0.189 | -4.363 | <.001 | -1.198 | -0.455 |
| N = sample size used in the analysis; β = standardized linear regression coefficient; SE (β) = standard error of the standardized linear regression coefficient; Z = Z-test of the intercept or of the regression coefficient; CI = confidence intervals; * = p<0.05. | | | | | | | |

*Table S8:Akaike Information Criterion (AIC) Values in the Fixed and Mixed-Effects Models (PHQ-9 Total Score (Linear Model))*

| **ORP Variables** | **PHQ-9 Total Score (Linear Model)** | | | | | | | |
| --- | --- | --- | --- | --- | --- | --- | --- | --- |
|  | **Fixed-Effects** | **Mixed-Effects model (Random Intercept)** | **Mixed-Effects model (Random Intercept and Random Slope for the ORP variable)** | | | **Mixed-Effects model (Random Intercept, Random Slope for the ORP variable, and Random Slope for the Covariates)** | | |
|  |  |  | **Full Covariance Matrix** | **Independent Covariance Matrix** | **Diagonal Covariance Matrix** | **Full Covariance Matrix** | **Independent Covariance Matrix** | **Diagonal Covariance Matrix** |
| avg orp all stages | 5155.049 | 5157.060 | 5178.957 | 5175.648 | 5209.820 | 5208.146 | 5269.444 | 5273.293 |
| avg orp N1+N2 | 5156.423 | 5158.438 | 5182.032 | 5175.242 | 5208.637 | 5209.437 | 5269.955 | 5272.919 |
| avg orp N3 | 4894.142 | 4895.737 | 4908.587 | 4911.265 | 4908.600 | 4949.816 | 4970.048 | 5001.554 |
| avg orp rem | 4848.487 | 4850.489 | 4898.613 | 4870.877 | 4898.613 | 4966.644 | 4921.326 | 4966.644 |
| avg orp wake | 5165.933 | 5167.944 | 5179.831 | 5181.921 | 5206.771 | 5222.084 | 5242.582 | 5271.184 |
| std dev all stages | 5157.108 | 5159.209 | 5195.114 | 5171.589 | 5195.114 | 5217.786 | 5259.487 | 5263.201 |
| std dev N1+N2 | 5164.979 | 5166.987 | 5178.555 | 5187.245 | 5204.735 | 5226.820 | 5269.137 | 5268.590 |
| std dev N3 | 4899.598 | 4901.973 | 4912.974 | 4927.395 | 4939.880 | 4966.884 | 5009.109 | 5004.447 |
| std dev rem | 4851.308 | 4853.308 | 4863.328 | 4877.714 | 4887.828 | 4909.093 | 4930.436 | 4955.455 |
| std dev wake | 5159.300 | 5161.600 | 5171.817 | 5188.018 | 5197.631 | 5222.038 | 5235.601 | 5260.182 |

*Table S9: Akaike Information Criterion (AIC) Values in the Fixed and Mixed Effects (PHQ-9 >= 10 (Logistic Model))*

| **ORP Variables** | **PHQ-9 >= 10 (Logistic Model)** | | | | | | | |
| --- | --- | --- | --- | --- | --- | --- | --- | --- |
|  | **Fixed-Effects** | **Mixed-Effects model (Random Intercept)** | **Mixed-Effects model (Random Intercept and Random Slope for the ORP variable)** | | | **Mixed-Effects model (Random Intercept, Random Slope for the ORP variable, and Random Slope for the Covariates)** | | |
|  |  |  | **Full Covariance Matrix** | **Independent Covariance Matric** | **Diagonal Covariance Matrix** | **Full Covariance Matrix** | **Independent Covariance Matric** | **Diagonal Covariance Matrix** |
| avg orp all stages | 1024.855 | 1079.743 | 1095.537 | 1094.962 | 1132.268 | 1129.130 | 1207.138 | 1200.882 |
| avg orp N1+N2 | 1026.243 | 1081.175 | 1098.129 | 1095.673 | 1131.222 | 1132.853 | 1160.412 | 1199.605 |
| avg orp N3 | 964.644 | 1017.209 | 1027.269 | 1031.493 | 1061.292 | 1070.096 | 1130.212 | 1131.709 |
| avg orp rem | 965.426 | 1017.845 | 1035.419 | 1034.888 | 1070.440 | 1064.977 | 1096.508 | 1137.955 |
| avg orp wake | 1035.607 | 1090.645 | 1104.182 | 1103.154 | 1135.457 | 1142.635 | 1169.751 | 1203.998 |
| std dev all stages | 1028.543 | 1083.773 | 1096.258 | 1096.550 | 1124.181 | 1174.940 | 1166.305 | 1195.103 |
| std dev N1+N2 | 1036.652 | 1091.532 | 1103.608 | 1112.273 | 1131.184 | 1184.047 | 1167.290 | 1199.703 |
| std dev N3 | 968.762 | 1020.889 | 1032.334 | 1045.928 | 1060.071 | 1084.151 | 1104.821 | 1129.368 |
| std dev rem | 966.820 | 1018.954 | 1029.312 | 1046.650 | 1056.527 | 1107.444 | 1105.915 | 1108.506 |
| std dev wake | 1030.993 | 1085.818 | 1097.064 | 1110.838 | 1125.269 | 1146.839 | 1167.824 | 1193.905 |
